# Simulating mismatch between calibration and target population in AI for mammography the retrospective VAIB study

**DOI:** 10.1038/s41746-025-01623-0

**Published:** 2025-05-08

**Authors:** Haiko Schurz, Klara Solander, Davida Åström, Fernando Cossío, Taeyang Choi, Magnus Dustler, Claes Lundström, Håkan Gustafsson, Sophia Zackrisson, Fredrik Strand

**Affiliations:** 1 Department of Oncology-Pathology, Karolinska Institutet, Solna, Sweden; 2 Medical Diagnostics Karolinska, Karolinska University Hospital, Solna, Sweden; 3 Department of Translational Medicine, Diagnostic Radiology, Lund University, Malmö, Sweden; 4 Center for Medical Image Science and Visualization (CMIV), Linköping University, Linköping, Sweden; 5 Sectra AB, Linköping, Sweden; 6 Department of Medical Radiation Physics, and Department of Health, Medicine and Caring Sciences, Linköping University, Linköping, Sweden; 7 Department of Imaging and Physiology, Skåne University Hospital Malmö, Malmö, Sweden

**Keywords:** Machine learning, Quality control, Statistical methods

## Abstract

AI cancer detection models require calibration to attain the desired balance between cancer detection rate (CDR) and false positive rate. In this study, we simulate the impact of six types of mismatches between the calibration population and the clinical target population, by creating purposefully non-representative datasets to calibrate AI for clinical settings. Mismatching the acquisition year between healthy and cancer-diagnosed screening participants led to a distortion in CDR between −3% to +19%. Mismatching age led to a distortion in CDR between −0.2% to +27%. Mismatching breast density distribution led to a distortion in CDR between +1% to 16%. Mismatching mammography vendors lead to a distortion in CDR between −32% to + 33%. Mismatches between calibration population and target clinical population lead to clinically important deviations. It is vital for safe clinical AI integration to ensure that important aspects of the calibration population are representative of the target population.

## Introduction

In recent years AI systems have made significant advances in breast cancer detection performance^1–8^ and there are multiple FDA-approved and CE-marked commercially available systems for clinical use^9^^,^^10^. Prospective trials have shown the significant impact of integrating AI in the screening workflow with the potential to increase cancer detection and reduce the workload compared to double reading by radiologists^7,8,11^. In the ScreenTrustCAD study^11^ single reading by AI and double reading by AI plus one radiologist was compared to the standard radiologist double reading workflow. The results from the 55,581 screening participants included in the trial show that double reading by AI plus one radiologist led to a 4% increase in cancer detection and a 50% reduction in radiologist workload. In the randomized, controlled MASAI trial AI was used to triage screening mammograms in the 40,000-woman-study group to be read by either one or two radiologists^7^. This implementation led to an increase in cancer detection while maintaining the false positive rate and reducing the radiologist workload by 44% compared to the standard double reading by radiologist. Finally, the prospective study by Annie et al. (2023) used AI as an additional reader to the double reader setting to flag cases for further arbitration review^8^. This resulted in a 5–13% increase in cancer detection rate per 1000 screening examinations at only a marginal, 0–0.23%, increase in unnecessary recalls^8^^,9^.

These prospective trials have demonstrated that AI systems may improve mammography screening, at least in the specific settings in which the trials were conducted. Currently, there are multiple examples of AI models which performed well in one dataset but failed to maintain this performance when used in a clinical setting or validated on a completely independent and external dataset^10,12–14^. Rigorous prospective studies in specific settings are therefore needed. However, trials are very costly and time-consuming and thus not a viable prerequisite for every combination of AI systems, mammography systems and each specific clinical setting. Furthermore, as the number of commercially available AI systems increases rapidly, a method for choosing the best algorithm for a specific clinical setting is needed. For this purpose, we have established the validation for AI in breast imaging (VAI.B) platform through which we collected an extensive number of screening mammograms from three regions across Sweden^15^. VAI.B is a collaboration between Swedish research groups, hospitals, and regional cancer centers, which aims to leverage retrospective data to validate, calibrate and compare AI systems for clinical use.

We demonstrate the importance of proper external validation methodology for AI systems and calibration of the AI systems output. We investigated a panel of validation errors resulting from differences between the population used to validate and calibrate the AI system compared to the target setting where the AI is to be implemented. Assuming an implementation where AI replaces radiologists, we first determined, for each AI system, the expected cancer detection rate and false positive rate when calibrating the AI threshold in a representative dataset, and then determined how those would increase or decrease depending on if the threshold had been determined in a non-representative dataset in terms of e.g., age, density and examination year.

## Results

### Study population

A total of 7790 exams from 6028 cancer-diagnosed participants and 38,509 exams from 27,422 healthy participants were included in the final case-control dataset. The cohort data had a total of 867 exams from 864 cancer-diagnosed participants and 40,022 exams from 39986 healthy screening participants (Table 1). A flowchart of the data quality control process is shown in Supplementary Fig. 1.

**Table 1:** Summary of the number of screening participants and examinations, for cancer-diagnosed cases and healthy controls, for the entire dataset and relevant subgroups

Group	Subgroup	Case-Control				Cohort			
		Participants		Exams		Participants		Exams	
		Case	Control	Case	Control	Case	Control	Case	Control
Overall	Overall	6028	27,422	7790	38,509	864	39,986	867	40,022
Image acquisition (equipment manufacturer)	GE	3692 (61.3%)	19,868 (80.6%)	5049 (65%)	30,730 (80%)	491 (57.2%)	20,598 (53.1%)	493 (57.3%)	20,369 (51.9%)
	Philips	2327 (38.7%)	4776 (19.4%)	2723 (35%)	7663 (20%)	367 (42.8%)	18,166 (46.9%)	368 (42.7%)	18,868 (48.1%)
Population characteristics: Age	40–49	1037 (16.5%)	8386 (28.6%)	1257 (16.1%)	9362 (24.3%)	146 (16.9%)	11,702 (29.3%)	147 (17%)	11,706 (29.2%)
	50–69	3885 (61.7%)	16,747 (57.2%)	4886 (62.5%)	23,540 (61.1%)	536 (62%)	21,884 (54.7%)	538 (62%)	21,908 (54.7%)
	70+	1371 (21.8%)	4159 (14.2%)	1677 (21.4%)	5608 (14.6%)	182 (21.1%)	6404 (16%)	182 (21%)	6415 (16.1%)
	Pre-menopause^a^	1826 (30%)	13,241 (47%)	2257 (29%)	15,393 (40%)	261 (30%)	18,642 (44.6%)	261 (30%)	18,650 (46.6%)
	Post-menopause^b^	4277 (70%)	14,834 (53%)	5495 (71%)	23,058 (60%)	603 (70%)	21,344 (55.4%)	603 (70%)	21,372 (53.4%)
Population characteristics: Mammographic density^c^	A	582 (9.3%)	4139 (14.6%)	677 (7.9%)	5478 (14.2%)	88 (10.2%)	5700 (14.3%)	88 (9.6%)	5700 (14.2%)
	B	3313 (52.6%)	14,958 (52.8%)	4187 (48.6%)	20,568 (53.4%)	450 (52%)	20,570 (51.4%)	499 (54.5%)	20,591 (51.4%)
	C	2219 (35.3%)	9038 (31.9%)	2732 (31.7%)	11207 (29.1%)	305 (35.3%)	12,613 (31.5%)	307 (33.5%)	12,621 (31.6%)
	D	176 (2.8%)	194 (0.7%)	1013 (11.8%)	1259 (3.3%)	22 (2.5%)	1117 (2.8%)	22 (2.4%)	1119 (2.8%)
Cancer characteristics	Invasive	4990 (86.7%)	NA	6462 (86.7%)	NA	708 (85.3%)	NA	710 (85.3%)	NA
	In situ	768 (13.3%)	NA	995 (13.3%)	NA	122 (14.7%)	NA	122 (14.7%)	NA

^a^Pre-menopause is defined as screening participants less than or equal to 55 years of age.

^b^Post-menopause is defined as screening participants more than 55 years of age.

^c^Density categories were assigned by the Lunit INSIGHT MMG system (South Korea, v.1.1.7.2).

### Validation errors

For each validation error the AUC and clinical impact, in terms of CDR and FPR, was calculated and compared for the overall VAI.B data compared to the incorrect, validation error, data selection. Summary statistics for each validation error are provided in Table 2 and the complete evaluation metrics for all validation errors are provided in the supplementary data.

**Table 2:** Summary of the impact of the validation error data selection on the overall AI performance (AUROC) and impact on CDR and FPR when used for AI threshold calibration

Characteristic	Representative population	Calibration bias	AUROC *p*-value	% change in CDR estimate	% change in FPR estimate
1. Reference standard (follow-up time)	3-year follow-up	1-year follow-up	<0.001	NA	
		2-year follow-up	<0.001		
		4-year follow-up	<0.001		
2. Examination year	Representative selection for each calendar year (2008–2019)	Early cases (2008–2012) and late controls (2015–2019)	<0.001	−2.7 to +1.1	−13.3 to +5.9
		Early control (2008–2012) and late cases (2015–2019)	<0.001	+17.3 to +19.2	+86.9 to +130
3. Population characteristics: Age at examination	Representative age distribution (40–74)	Post-menopause cases (>55) and pre-menopause controls (≤55)	<0.04	−0.2 to +3.8	−0.2 to +18.8
		Pre-menopause cases (<=55) and post-menopause controls (>55)	<0.001	+15.5 to +26.8	+95 to +159.5
4. Population characteristics: Mammographic density	Representative density distribution	Density A and B only	<0.02	+1.1 to +6.9	+4 to +35.7
		Density C and D only	<0.001	+8.3 to +15.8	+44.3 to +80.4
		Density A, B and C only	<0.5	+6.2 to +6.8	+26.9 to +35.1
5. Cancer characteristics	Representative distribution	Invasive only	<0.06	+7.9 to +9.3	+38.2 to +41.8
		In situ only	<0.08	−8.9 to +11.6	−8.9 + 11.6
6. Image acquisition (equipment manufacturer)	GE only	Philips only	<0.001	−13.1 to −32.5	−39.2 to −78.2
	Philips only	GE only	<0.001	+10.6 to +33.4	+62.9 to +442
	Representative GE and Philips	GE case and Philips controls	<0.001	+11.6 to +20.3	+59.1 to +137.0
		Philips case and GE control	<0.001	−3.8 to −18.4	−8.1 to −59.8

The minimum to maximum percent change across all three AI systems is provided for CDR and FPR with a negative value indicating percent reduction and positive values indicating percent (%) increase.

*AUROC* Area under the receiver operating curve, *CDR* Cancer detection rate, *FPR* False positive rate.

### Follow-up period and reference standard

**Fig. 1:**
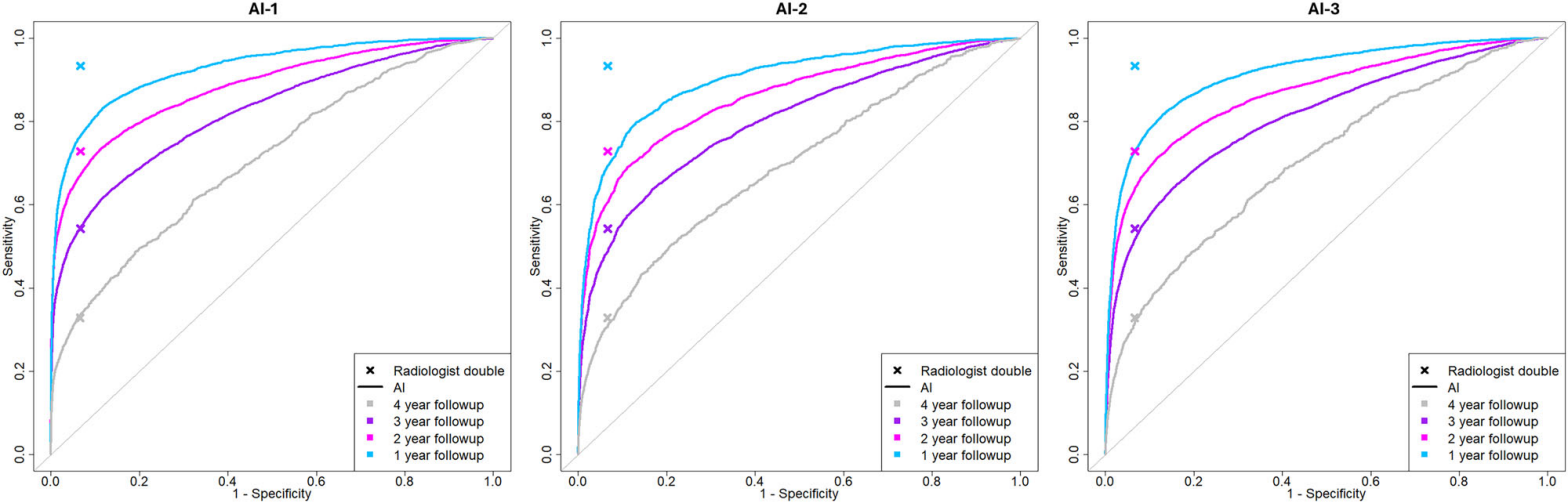
Follow-up period and reference standard—comparing radiologists double reading to AI systems. Impact of different follow-up times as the basis for the reference standard for AI performance compared to radiologists double reading for the three AI systems. As the follow-up period is shortened the bias in favor of the radiologist increases (the ‘x’ denoting radiologist performance is further away from the ROC curve of AI). Even if the overall accuracy is lower when using a 3-year (purple) follow-up period since cancer diagnosis is more distant from the time of the mammography, it is the shortest timeframe to avoid bias in favor of radiologist compared to AI detection.

To compare the performance of the AI systems to the screening performance of the original radiologists for different follow-up periods (reference standards to define cancer cases and healthy control) we set the follow-up period for the reference standard to either 12, 24, 36 or 48 months. The radiologist sensitivity (at 93% specificity) for the 48-, 36-, 24- and 12-month follow-up period was 33% (95% CI, 32.2–33.7), 54% (95% CI, 53.1–55.4), 73% (95% CI, 71.6–74.2) and 93% (95% CI, 92.4–94.2) respectively. As shown in Fig. 1 and Supplementary Fig. 2 the performance of the radiologist is in line with the AI performance, for all three AI systems, when the follow-up period was set to 36 months between exam and diagnosis. As the follow-up period is shortened the radiologist consistently outperformed the AI systems and as a result the AI systems could not match this performance without significantly inflating the FPR.

### Temporal selection

To demonstrate the importance of a representative selection of data for each examination year we selected cases and controls from different time periods. When selecting cases from early years (2008–2010) and controls from later years (2017–2019) the performance (AUC) of all three AI systems was significantly inflated compared to the representative dataset (*p*-value < 0.001; Supplementary Fig. 3 and Supplementary Fig. 4). Selecting controls from early years (2008–2010) and cases from later years (2017–2019) significantly deflated the AI performance (AUC) compared to the representative dataset (*p*-value < 0.001; Supplementary Fig. 3 and Supplementary Fig. 4).

To further demonstrate the impact of non-representative data selection we used the non-representative data to calibrate the AI systems (to radiologists double reading CDR) and then applied this threshold to the overall, representative, dataset. Using the threshold from the early cases and late controls reduces the CDR and FPR for AI-1 (2.7% CDR: 95% CI, 2.3–3.3; 13.3% FPR: 95% CI, 13.2–13.5) and AI-3 (0.2% CDR: 95% CI, 0.1–0.4; 0.3% FPR: 95% CI, 0.1–0.34) while increasing it for AI-2 (1.1% CDR: 95% CI, 0.9–1.6; 5.9% FPR: 95% CI, 4.6–7.2; Supplementary Table 1). Conversely when calibrating the representative data using the early controls and late cases threshold then the CDR and FPR is increased for AI-1 (19.2%CDR: 95% CI, 11.6–25.3, 130% FPR: 95% CI, 129–131), AI-2 (17.3%CDR: 95% CI, 11.0–23.3, 86.9% FPR: 95% CI, 85.6–87.7), and AI-3(19.2%CDR: 95% CI, 18.1–22.8, 102% FPR: 95% CI, 101–103; Supplementary Table 1). The main factors contributing to the changes in performance are the differences in the distribution of mammography imaging equipment manufacturer models and software versions between the early and late data (Supplementary Table 2).

### Population characteristics: age and mammographic density

All three AI systems perform significantly better (*p*-value < 0.001) for older participants (over 70) compared to younger age groups (40–49) [Supplementary Figs. 5, 6 and 7]. Using an unrepresentative validation dataset to calibrate the threshold for AI systems has a significant impact on the accuracy metrics when applied to the representative dataset containing the correct age distribution (Supplementary Table 3). Using data containing only the age range of 40–49 to calibrate the AI inflated the CRD and FPR values when applied to the overall dataset (**AI-1**: 19.1% CDR: 95% CI, 17.9–20.4 and 129.5% FPR: 95% CI, 128.4–130; **AI-2** 36.2% CDR: 95% CI, 34.1–37.5 and 232% FPR: 95% CI, 230–233; **AI-3** 31.6% CDR: 95% CI, 30.1–31.9 and 200.8% FPR: 95% CI, 199–202; Supplementary Table 3). Similarly using age range 50–69 also inflated the CRD and FPR values (**AI-1**: 7.3% CDR: 95% CI, 6.3–8.3 and 37% FPR: 95% CI, 36.7–37.4; **AI-2** 6.1% CDR: 95% CI, 5.8–6.9 and 26.1% FPR: 95% CI, 25.7–26.2; **AI-3** 6.7% CDR: 95% CI, 5.5–7.5 and 29.6% FPR: 95% CI, 29.4–30.1; Supplementary Table 3), while using age range 70–74 deflated the CDR and FPR values when applied to the representative dataset (**AI-1**: 2% CDR: 95% CI, 1.9–2.1 and 11.2% FPR: 95% CI, 11.0–11.4, **AI-2** 8.7% CDR: 95% CI, 8.3–9.1 and 22.7% FPR: 95% CI, 22.6–22.8, **AI-3** 8.3% CDR: 95% CI, 8.2–8.6 and 25.9% FPR: 95% CI, 25.7–26.1; Supplementary Table 3).

To demonstrate the impact of non-representative age selection, the AI threshold was determined using the non-representative age dataset and applied to the overall, representative, dataset. Using post-menopause cases (>55) and pre-menopause controls (<=55) significantly inflated overall AI performance for AI-2 and AI-3 (*p*-values < 0.001) but did not have a significant impact on AI-1 (*p*-value = 0.18, 5 and Supplementary Table 4). When combining pre-menopause cases and post-menopause controls the overall AI performance for all three systems was significantly deflated (*p*-value < 0.001, Supplementary Fig. 5 and Supplementary Table 4). Using the post-menopause cases and pre-menopause controls to calibrate the AI threshold led to inflated CDR and FPR values for AI-1 and AI-3 (**AI-1**: 3.8% CDR: 95% CI, 3.7–4.3 and 18.8% FPR: 95% CI, 18.6–19.2, **AI-3** 0.2% CDR: 95% CI, 0.1–0.3 and 0.3% FPR: 95% CI, 0.1–0.5; Supplementary Table 4) and slightly deflated CDR and FPR values for AI-2 (**AI-2** 0.2% CDR: 95% CI, 0.1–0.4 and 0.2% FPR: 95% CI, 0.1–0.3; Supplementary Table 4). Calibrating using the pre-menopause cases and post-menopause controls inflated the CDR and FPR when applied to the overall dataset (**AI-1**: 15.5% CDR: 95% CI, 15.3–15.8 and 95% FPR: 95% CI, 94.4–95.6, **AI-2** 26.8% CDR: 95% CI, 26.3–27.1 and 159.5% FPR: 95% CI, 159–160, **AI-3** 25% CDR: 95% CI, 23.6–26.4 and 144.3% FPR: 95% CI, 143–145; Supplementary Table 4).

As breast density is inversely correlated with age (breast density for most women decreases with age) we investigated the impact of mammographic density on AI performance^16,17^. All three AI systems have a significant difference in performance between mammographic density A and D (*p*-value < 0.001) and perform best on breasts with low mammographic density (category A) and worst on breasts with high mammographic density (category D, Supplementary Fig. 8, Supplementary Fig. 9). However, as shown in Supplementary Fig. 8, the impact of removing mammographic density category D from the data does not have a significant impact on overall AI performance (Supplementary Fig. 8, Supplementary Fig. 10). This is due to the low number of participants with mammographic density category D (194 cases and 1013 controls). However, while the overall performance is not significantly impacted (*p*-value = 0.4), not including mammographic density category D does shift the distribution of AI scores. When the AI is calibrated without considering mammographic density category D and then applied to the overall data it leads to inflated CDR and FPR values (**AI-1**: 6.8% CDR: 95% CI, 5.7–7.0 and 35.1% FPR: 95% CI, 34.9–35.4, **AI-2** 6.2% CDR: 95% CI, 5.8–6.7 and 26.9% FPR: 95% CI, 26.8–27.0, **AI-3** 6.2% CDR: 95% CI, 5.5–6.5 and 28% FPR: 95% CI, 27.8–28.3; Supplementary Table 5). Similarly, limiting the validation data to only low mammographic density (category A and B) or high mammographic density (category C and D) also skews the overall performance (*p*-value < 0.001) of the AI systems (Supplementary Fig. 8). Calibrating the AI on low mammographic density categories inflates the CDR and FPR values when applied to the overall dataset (**AI-1**: 6.9% CDR: 95% CI, 5.9–7.2 and 35.7% FPR: 95% CI, 35.6–35.9, **AI-2** 3.3% CDR: 95% CI, 3.1–3.5 and 14.2% FPR: 95% CI, 13.6–14.7, **AI-3** 1.1% CDR: 95% CI, 0.08–1.3 and 4% FPR: 95% CI, 3.9–4.1; Supplementary Table 5). Applying the threshold from the high mammographic density categories to the overall dataset also inflates the CRD and FPR values when applied to the overall dataset (**AI-1**: 8.3% CDR: 95% CI, 7.5–8.9 and 44.3% FPR: 95% CI, 44.0–44.5, **AI-2** 14.11% CDR: 95% CI, 13.5–14.5 and 72.1% FPR: 95% CI, 71.7–72.3, **AI-3** 15.8% CDR: 95% CI, 15.1–16.0 and 80.4% FPR: 95% CI, 80.2–80.6; Supplementary Table 5).

### Cancer characteristics

For AI-1 there was no significant difference in AUC values between the overall dataset, in-situ and invasive subgroups (*p*-value > 0.07, Supplementary Fig. 11, Supplementary Fig. 12). For AI-2 there was a significant difference in AUC values between the overall and in-situ and the in-situ and invasive subgroups (*p*-value < 0.001, Supplementary Fig. 12). AI-3 had significant differences in performance between the in-situ and invasive subgroups AUC values (*p*-value < 0.001, Supplementary Fig. 12).

Using data containing only in-situ cancers to calibrate the AI threshold and applying it to the overall dataset led to an increase in CDR and FPR for AI-1 (2.5% CDR: 95% CI, 1.7–2.8 and 11.6% FPR: 95% CI, 11.4–11.8) and a decrease in CDR and FPR for AI-2 (4.4% CDR: 95% CI, 4.1–4.8 and 8.9% FPR: 95% CI, 8.8–9.0) and AI-3 (1.5% CDR: 95% CI, 1.0–2.4 and 4.5% FPR: 95% CI, 4.4–4.6; Supplementary Table 6). Calculating the threshold using invasive data only led to an increase in CDR and FPR for all AI systems (**AI-1**: 7.9% CDR: 95% CI, 8.6–7.1 and 41.8% FPR: 95% CI, 41.0–42.6, **AI-2** 8.6% CDR: 95% CI, 8.0–9.3 and 41.1% FPR: 95% CI, 40.9–41.4, **AI-3** 9.3% CDR: 95% CI, 8.9–9.7 and 38.2% FPR: 95% CI, 37.9–38.5; Supplementary Table 6).

### Image acquisition

**Fig. 2:**
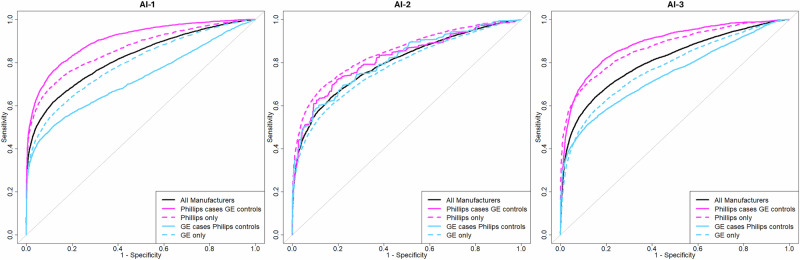
ROC of image acquisition equipment Philips and GE. ROC curve for the AI performance on different mammography imaging equipment manufacturers (dashed lines) and case-control selection from different manufacturers (solid lines) compared to the overall dataset containing exams from both equipment manufacturers.

AI performance on different imaging equipment manufacturers, GE and Philips, was assed. Figure 2 demonstrates that the AI systems perform significantly better on Philips compared to GE images (*p*-value < 0.001, Supplementary Fig. 13 and 14). Using images from Philips equipment to calibrate the AI systems for GE images reduces CDR and FPR values for all three AI systems (**AI-1**: 30% CDR: 95% CI, 29.2–30.8 and 78.2% FPR: 95% CI, 77.9–78.4, **AI-2** 13.1% CDR: 95% CI, 11.9–13.5 and 39.2% FPR: 95% CI, 39.1–39.4, **AI-3** 32.52% CDR: 95% CI, 32.0–33.3 and 70.2% FPR: 95% CI, 70.1–70.4; Supplementary Table 7). Alternatively using GE images to determine the AI calibration threshold for Philips images results in a significant increase in CDR and FPR for all three AI systems (**AI-1**: 33.4% CDR: 95% CI, 31.2–34.6 and 442% FPR: 95% CI, 437–447, **AI-2** 10.6% CDR: 95% CI, 9.8–11.4 and 62.9% FPR: 95% CI, 62.5–63.7, **AI-3** 33% CDR: 95% CI, 31.5–34.0 and 436% FPR: 95% CI, 429–441; Supplementary Table 7). As the screening participant distributions (in terms of the cancer diagnosis and healthy ratio, density and age, Supplementary Table 9) are similar across all the sites, the screening participant characteristics are not the main factor for these performance differences.

To further demonstrate the significant impact that data selection from different equipment manufacturers can have we skewed the ratio of equipment manufacturers for cases and control in the data. When using cancer cases from Philips and healthy controls from GE there is a significant inflation in overall AI performance (*p*-value < 0.001, Supplementary Fig. 14). Using cancer cases from GE and controls from Philips leads to significantly reduced AI performance (*p*-value < 0.001, Fig. 2, Supplementary Figs. 13 and 14). When the non-representative GE cases and Philips controls dataset were used to calibrate the AI systems it led to increased CDR and FPR values when applied to the overall representative dataset (**AI-1**: 20.3% CDR: 95% CI, 20.0–20.7 and 137% FPR: 95% CI, 136.7–137.3, **AI-2** 11.6% CDR: 95% CI, 10.5–12.2 and 59.1% FPR: 95% CI, 58.8–59.4, **AI-3** 20.1% CDR: 95% CI, 19.8–20.4 and 106.7% FPR: 95% CI, 105.8–106.9; Supplementary Table 8). Conversely when the GE controls and Philips cases data was used to calibrate the AI for the overall dataset it led to decreased CDR and FPR values (**AI-1**: 16.9% CDR: 95% CI, 16.2–17.6 and 59.8% FPR: 95% CI, 59.6–60.0, **AI-2** 3.8% CDR: 95% CI, 4.2–3.4 and 8.1% FPR: 95% CI, 7.9–8.3, **AI-3** 18.4% CDR: 95% CI, 17.6–18.5 and 50% FPR: 95% CI, 49.9–50.2; Supplementary Table 8).

To further highlight the importance of using local data to validate we demonstrated the differences in performance of the AI systems across the three regions from which the data was collected (Supplementary Fig. 15, Supplementary Fig. 16). As the different regions have different equipment manufacturers it is to be expected that Östergötland, which uses Philips performs significantly better (*p*-value < 0.001) compared to Södermanland and Västmanland, which use GE. However, when comparing the performance of the AI systems for the two regions that use GE equipment (Södermanland and Västmanland), we still observed significant differences in overall AI performance (*p*-value < 0.001, Supplementary Fig. 15, Supplementary Fig. 17). A probable driving factor for the differences in AI performance between the two regions using GE equipment is the fact that the imaging equipment manufacturers and software versions are not balanced in the cases and controls between these regions (Supplementary Table 10).

### Statistical analysis and reporting metrics

Results indicate that both upscaling and bootstrapping are viable options for adjusting the accuracy metrics for the case-control dataset. Upscaling and bootstrapping provide very similar results compared to the cohort data, showing only minor differences in the accuracy metrics, both in terms of the point estimates and 95% confidence intervals (Supplementary Table 11). However, the confidence intervals are generally wider for the bootstrapping method compared to upscaling.

To compare the difference between 1:5 case-control ratio and 1:1 case-control ratio we randomly selected controls from the 1:5 case-control data to make a 1:1 case-control dataset. Overall, the results for the 1:5 and 1:1 ratio dataset are very similar for both the upscaling and bootstrapping methods. However, the 95%CI for the 1:1 ratio data is wider compared to the 1:5 ratio data (Supplementary Table 11).

## Discussion

The results demonstrate that multiple data selection and calibration validation errors can have clinically significant impact when planning for use of AI in screen-reading and hence have the potential to be problematic for breast cancer screening programs. All the validation errors investigated have a significant impact on the ROC-AUC, calibration threshold or both, which results in significant changes in cancer detection rate and false positive rate. Equipment manufacturer differences had the largest impact on AI performance for all three algorithms with significant differences in ROC-AUC and up to 33% (95% CI, 31.5–34.0) change in cancer detection rate and 442% (95% CI, 437–447) change in false positive rate. These results highlight the importance of doing local validation and calibration using data that reflects the target clinical setting.

Evaluation of AI systems requires a defined reference standard (ground truth) to assess AI performance^18,19^. The reference standard establishes the presence or absence of the target condition, in our case the presence or absence of breast cancer diagnosis. Since it cannot be known with certainty if currently healthy participants will develop cancer in the future, there is a need to define a reasonable reference standard, based on a specified follow-up period.

Based on the results there is a significant bias towards the radiologists for a 12- and 24-month follow-up. At a 36-month follow-up, the AI performance for all three AI systems is in line with the radiologist's double reading performance. When calibrating and validating AI systems for a clinical setting where the purpose is to match the performance of the radiologist it is vital to choose an appropriate follow-up period where the performance is not biased in favor of the radiologists. While the results indicate that the definition of the reference standard is highly relevant to the evaluation of AI systems, many previous studies did not consider this fact^5,20–23^. Previous publications explain the importance of using the correct accuracy metrics when evaluating AI systems but fail to elaborate on the fact that these accuracy metrics are dependent on the reference standard. Likewise, publications evaluating AI models choose either a 12- or 24-month follow-up period without providing evidence that this is the optimal reference or considering alternative reference periods^1,2,5,6,24^. In addition to this, previous studies assessing AI performance combine results from studies using different reference periods, which can introduce bias into the results and paint an inaccurate picture of AI performance. For example, a systematic review by Freeman et al.^6^ concluded that 94% of AI systems evaluated were less accurate compared to radiologists^6^. However, with a 12-month follow-up, these results are likely due to the bias towards the radiologist introduced using a short follow-up period in the reference standard. Careful consideration of the follow-up period is especially important when data from countries with different screening intervals is combined for external AI validation. The multicenter study by Rodriguez-Ruiz et al.^24^ combined data from various sources and while each dataset had at least one year follow-up it is not clear what the exact follow-up time for each dataset was. This mixture of follow-up times could have a strong impact on the performance of the AI system and lead to unclear results (Rodriguez-Ruiz et al.^24^). Future publications should assess the reference standard and follow-up more carefully to ensure both accurate and fair evaluation of AI systems.

When collecting retrospective data for the purposes of validation and calibration of AI systems prior to clinical implementation it is vital to balance the study dates and number of cases and controls over the entire time frame of interest to avoid temporal selection bias. This ensures that all clinical variables (imaging manufacturer, image post-processing, radiologists) are equally represented and allows for a fair evaluation of AI systems for a specific (and current) clinical setting.

Using a non-representative dataset results in skewed distributions in terms of imaging equipment manufacturer, manufacturer models and software versions between the cases and controls which has a significant impact on the AI performance compared to the representative dataset. This leads to significant changes in overall AI performance across all AI systems. Cancer detection rate varied between 19% (95% CI, 11.6–25.3) higher and 3% (95% CI, 2.3–3.3) lower than expected and false positive rate values varied between 130% (95% CI, 129–131) higher and 13.3% (95% CI, 13.2–13.5) lower than expected. Based on these results, calibrating an AI system using non-representative data that does not accurately reflect the clinical setting would result in an inaccurate calibration. This has the potential for a reduced cancer detection rate and an increased false positive rate which would result in increased workload for radiologists. Previous publications used retrospective data selected cases and randomly selected controls^1,2,5,6,24^. This would balance the ratio of cancer cases and controls overall but may skew the ratio for individual calendar years included in the data. While slight variations in case-control ratio between calendar years would not have a significant impact on the results, it would be good practice to ensure that the balance is maintained for validation to produce truly accurate results.

Screening participant age is an important factor to consider when validating AI systems for a clinical setting. First, there is an inverse correlation between age and breast density, with breast density decreasing as age increases^16,17^. Secondly, the risk of breast cancer increases with age^25,26^. Finally, the age range of the screening population differs from country to country based on the local screening program. For example, the Swedish screening program invites participants for screening between the ages of 40–74^27^, while the British screening program invites participants between the ages 50–70^28^.

We investigated the performance of the AI systems for different age groups and the results show a clear difference in overall AI performance, with the performance significantly increasing for the older age groups. Furthermore, using non-representative data or data restricted to a limited age that does not represent the screening population changes the CDR and FPR values. cancer detection rate varied between 27% (95% CI, 26.3–27.1) higher and 0.2% (95% CI, 0.1–0.4) lower than expected and false positive rate values varied between 159.5% (95% CI, 159–160) higher and 0.2% (95% CI, 0.1–0.3) lower than expected. Previous studies using cohort data or randomly selected screening participants to evaluate AI systems will maintain the age distribution and if the sampled population is representative of the screening population validation will produce accurate results^1,2,5,6,24^. However, if external validation data is sourced from a screening program with a different age range the AI performance may not be accurate to the local setting. This is especially important if multiple datasets are combined. In a previous study by Rodriguez-Ruiz et al. (2018), data was sourced from multiple counties, some of which have different screening programs, and used to assess AI performance compared to radiologists^24^. As the performance of AI is affected by age this comparison may be influenced by the mismatched age ranges between the datasets and adjusting for this in the analysis should be considered.

Ensuring that the validation data is reflective of the age distribution is vital for clinical validation. This is especially important when merging multiple datasets that may have different selection criteria or originate from a country with a different screening population age. The distribution of age (and breast density) should always be compared to the actual screening population to allow for accurate validation and calibration of AI systems.

AI systems and radiologists perform best on the lowest mammographic density A and worst on mammographic density D, which is in line with previous publications^29–32^. Due to the variable performance on different breast densities, it is important for any validation dataset to reflect the breast density distribution of the target population. For the VAI.B platform, we used random selection to ensure that our case-control retrospective dataset reflects the correct distribution of breast densities.

Overall, the results indicate that there are significant differences in AI performance between low (A and B) and high (C and D) densities. When removing the highest mammographic density (category D) from the data the overall AI performance is not significantly altered. The reason for this is that the number of screening participants with mammographic density D breasts is not enough to alter the overall AI performance. However, while the overall AI performance is not significantly impacted, removing mammographic density category D from the data does shift the AI score distribution. This shift leads to a 6.2% (95% CI, 5.8–6.7) to 6.8% (95% CI, 5.7–7.0) increase in cancer detection rate and a 26.9% (95% CI, 26.8–27.0) to 35.1% (95% CI, 34.9–35.4) increase in false positive rate, when compared to the expected results. Previous publications either used continuous cohort datasets or randomly selected data and as such the mammographic density categories are representative of the screening population^1,2,5,6,24^. While previous publications have avoided this validation error through random selection it should be common practice to double-check that selected data has a representative mammographic density distribution. Furthermore, AI models that provide a mammographic density category could be extremely useful as radiologists can be alerted and take extra precautions evaluating high-density mammograms. Our results show that while mammographic density category D does not impact the overall performance the AI score distribution is impacted, and future research could investigate altering the AI score threshold for high mammographic density breasts.

Cancer characteristics are important factors to consider when evaluating AI systems. If an AI system performs extremely well for some cancer characteristics but poorly in other cancer characteristics it will create bias in the screening programs and lead to unfair treatment. As such it is vital to ensure that the full spectrum of cancer characteristics is represented in the validation data.

Between in-situ and invasive cancer cases we did not detect a consistent significant difference in performance across all three AI systems. However, there were shifts in the cancer detection rate and false positive rate when either in-situ or invasive-only data was used to calibrate the AI system for the representative completed dataset. The impact on cancer detection rate and false positive rate when invasive or in-situ data was used was not as extensive compared to other factors investigated in this work. Cancer detection rate varied between 9.3% (95% CI, 8.9–9.7) higher and 4.4% (95% CI, 4.1–4.8) lower than expected and false positive rate values varied between 41.8% (95% CI, 41.0–42.6) higher and 8.9% (95% CI, 8.8–9.0) lower than expected, with AI-1 being least affected. In this study, we are not able to determine the reason why AI-1 was impacted less by the cancer characteristics. One possible reason could be that the developers behind AI-1 have been able to make the system more consistently accurate across invasive and in situ cancers, possibly by having a better approach to train models on unbalanced data since there is usually a much smaller proportion of in situ cancer compared to invasive cancer in any population.

One factor that could influence this variation in performance for the cancer characteristics across the three AI systems is that only 13% of the cancer cases are diagnosed as in-situ cancers, which may not be enough to significantly shift the score distribution. However, there was still a detectable impact, which can have real-world consequences. Previous research using random selection or cohort data will automatically have representative cancer characteristics^1,2,5,6,24^, however, it should be common practice to check the cancer characteristics to ensure all diagnoses and subtypes are represented. Furthermore, the AI models should be evaluated on all subtypes to ensure no bias is introduced when the AI system is employed in a clinical setting. These factors include the tumor size, grade, ductal vs. lobular, receptor status as well as diagnosis type such as screen-detected and interval cancers.

Finally, imaging equipment manufacturers had by far the largest impact on AI performance. The equipment manufacturer is also the most variable factor within screening programs with equipment varying between and even within screening facilities. When validating and calibrating AI systems for a specific clinical setting it is thus vital for the validation data to be from the same imaging equipment.

Our results show a significant difference in performance between GE and Philips equipment for all three AI systems. Using the wrong equipment manufacturer to calibrate the AI systems led to cancer detection rate changes between 33.4% (95% CI, 31.2–34.6) higher and 32.5% (95% CI, 32.0–33.3) lower than expected and false positive rate values between 442% (95% CI, 437–447) higher and 78% (95% CI, 77.9–78.4) lower than expected. Furthermore, there were significant differences in performance between the two datasets from regions Södermanland and Västmanland that used GE imaging equipment. Distribution of screening participant characteristics is similar across the screening sites and thus do not account for these differences in performance. Potential contributing factors are differences in screening facilities, which may have different imaging settings and image post-processing, which result in variations in the final mammogram and subsequent variations in AI scores, as demonstrated in the study by Tingberg et al.^33^. To demonstrate the importance of calibrating representative data we used data containing only Philips images to calibrate AI systems for GE equipment and vice versa. Results show an extreme impact on cancer detection rate and false positive rate for all three AI systems. Previous studies have often limited the data to a single imaging equipment^1,2,30,31,34,35^. This is a good practice if the AI system is being evaluated for a specific equipment manufacturer, but extreme care should be taken when attempting to generalize these results to other imaging equipment manufacturers. Furthermore, care should be taken when performing meta-analysis which includes data from multiple sources as increased or decreased performance of the AI systems on specific equipment could skew the overall results^24^. Including appropriate subgroup analysis or regression to identify factors of heterogeneity in the meta-analysis should be common practice to highlight variables that impact AI performance^36^.

When validating AI systems, it is important for the results to reflect real-world cancer incidence rates to obtain realistic and clinically relevant cancer detection rates, abnormal interpretation rates, false positive rates, false negative rates, and positive predictive values^2^. For this reason, cohort datasets or prospective studies are generally preferred when validating AI systems for clinical use. However, collecting consecutive cohort data that contains enough cancer cases leads to extremely large datasets (especially if a longer time period is being considered), which are unrealistic to obtain for each clinical setting for which AI systems are to be evaluated as it requires immense data storage capacity and increased costs to run the AI systems on this data. These extensive costs are not feasible for resource-constrained breast screening institutions. Previous studies may have been penalized or scrutinized when using case-enriched case-control datasets to validate and evaluate AI systems. Our results however reveal that given stringent data collection procedures a case-control dataset combined with statistical methods (upscaling of healthy controls or weighted bootstrapping) to make the case-control ratio representative produces results that are statistically similar to cohort datasets. Using case-enriched data is thus a viable and less time-consuming and computationally intensive (both in terms of running and storage costs) method to accurately validate AI systems for clinical use.

The VAI.B cohort data, which contains all screening mammograms from 2017 contains 40896 examinations (867 cases and 40022 controls) compared to the case-control data set (limited to 2017 at a 1:5 case-to-control ratio), which contains 5201 examinations (867 cases and 4334 controls). Despite this significantly reduced dataset size both upscaling and weighted bootstrapping produce statistically similar point estimates and 95% confidence intervals for all accuracy measures when compared to the large cohort dataset. The results thus demonstrate that case enriched case-control dataset is a viable option for validating AI systems, especially when the function of the AI system is to detect cancer. The case-enriched data can include a larger number of cancer cases ensuring that the AI can be validated on all aspects of breast cancer characteristics.

In this manuscript, we highlight several validation errors and how to avoid them and why they are important considerations to make when validating AI systems. However, this work has some limitations. We used a 36-month follow-up period to define the reference standard, because of this we could not include the most recent data (2019–2022) that is available to us. Updated data from the cancer registry is required as AI validation and calibration should always be done on representative data that is current and relevant. While we covered several validation errors in this work this is by no means an exhaustive list and other factors that were not investigated here could have a similar impact. These factors include but are not limited to all cancer characteristics, ethnic background of screening participants (ethnic bias) equipment manufacturer model, software version, detector plate differences and differences in the settings used during image acquisition and image post-processing. Furthermore, we have not included a detailed analysis to highlight differences between AI models, in terms of exams that were successfully run on some AI systems but failed on others. Considering exams for which we only have inferences for a subset of the AI systems could highlight further discrepancies between the AI systems and how they respond to the validation errors. Another limitation is that while the same qualitative deviations can be expected in other settings, the specific magnitude of screening metric deviations is mainly applicable to the Swedish system or similar systems with biannual screening. Finally, we only included two imaging equipment manufacturers in our validation. An updated dataset including data from all commercially available imaging equipment manufacturers should be considered. The strengths of this manuscript are the large dataset we have available, which covers multiple screening facilities across Sweden and is thus a truly representative dataset with sufficient statistical power. Furthermore, we have both a case-enriched case-control dataset and a cohort dataset which was used to validate our statistical methods. Finally, even though we included three AI systems in our analysis of the impact of the validation errors to offer an insight into the distribution across AI systems, this does not mean that the magnitude of errors reported would be directly applicable to an AI system that was not included.

We conclude that proper validation methodology and representative data selection, which is representative of the target setting, are vital for the evaluation and safe integration of AI systems to be used in screening. Negligence in data selection and AI validation can have severe consequences when used for AI calibration, leading to significant changes in cancer detection rate and false positive rate. All validation errors investigated in this work had an impact on both overall AI performance and the cancer detection rate and false positive rate values when nonrepresentative data is used for calibration. This was especially evident when investigating the image equipment manufacturer, which had an extreme impact on the AI score threshold, which suggests that re-calibration of AI systems would be necessary for any screening equipment change. Implementing AI without these considerations and calibration could have extremely detrimental effects on the breast cancer screening program. This can potentially harm screening participants in terms of missed cancer detection or unnecessary worry and unnecessary medical procedures, which will result in an increased workload on an already overburdened healthcare system.

## Methods

### Study population

The VAI.B data was collected as described in previous work^15^. Ethical approval (EPM 2022–00186–01) was granted by the ethical review authority of Sweden to perform the scientific study, including the extraction of the data and its usage for evaluation purposes. The need for informed consent was waived by the ethics committee as it was deemed unfeasible and practically impossible to obtain informed consent retrospectively for such a large cohort of study participants. All personal information in the data was pseudonymized to protect the study participants’ identity. The data selection process for the case-control data was carried out as follows. From each of the three participating healthcare regions in Sweden (Östergötland, Västmanland, Södermanland), we first extracted from the Radiological Information System (RIS) screening registry the personal identification (ID) number, screening dates, and screening ID number of all women screened between January 1, 2008, and December 31, 2021. Participants diagnosed with breast cancer (both invasive and in situ), and the date of diagnosis, were ascertained by linking the personal ID number of the screening participant with data from the Swedish National Quality Register for Breast Cancer (NKBC). NKBC records every diagnosed breast cancer in Sweden and has national coverage, which means that even if the woman moved to a different healthcare facility any breast cancer diagnosis would still be included in our data.

NKBC data were extracted for breast cancer cases diagnosed between November 27, 2007, and April 22, 2022, and includes information on cancer diagnosis (invasive or in situ) and cancer pathology parameters which can be used for subgroup analyses. Healthy controls were defined as participants that had no record of breast cancer in the NKBC database over at least three years after the selected screening examination. For screening participants diagnosed with cancer all available mammography exams during the time period were included in the dataset and women with multiple diagnoses (28 participants) were not excluded. As the median screening interval in our data is 24 months, this includes the current and prior screening examinations for screen-detected cancers. For interval cancers (cancer diagnosed after a negative screening examination and before the next planned one) only the prior screening examination for most, except for 181 screening participants (diagnosed within 12 months of the most recent examination), is included.

The number of included controls was based on the number of participants diagnosed with cancers in each screening year between 2008 and 2021. The process for selection of healthy controls is outlined below:

1. All participants that had a screening examination were identified, and their status as healthy or diagnosed with cancer was ascertained (based on the NKBC record).
2. For each screening year, the number of cases with a mammography screening examination within 23 months prior to diagnosis was calculated.
3. Controls were chosen at random at a 1:5 ratio of cancer-diagnosed to healthy controls for each screening year in descending order 2021–2008.
4. For all selected participants (cases and controls), the mammography screening examinations in the screening year and within 5 years prior (if available) were extracted.
5. Selected screening participants of the specific screening year were then removed from the dataset and the steps, 1 to 3, were repeated for the next screening year (descending order).

From this data in the VAI.B database, a case-control dataset was established consisting of screening participants with breast cancer defined as cases and controls being women without breast cancer (based on a 36-month follow-up) at a ratio of 1:5 for each calendar year. Due to the three-year follow-up period, healthy status could only be confirmed for examinations acquired up to 3 years before the last NKBC record, limiting the latest time of the screening dates in the study population to April 2019. Thus, the final case-control dataset contains screening examinations between January 2008 and April 2019. For cancer cases, all exams within 36 months prior to diagnosis were included. For healthy controls, the most recent exam for each screening participant was included. In the case of duplicate screening views being present for a specific examination, we used the latest images to create a full set of the four standard view images to ensure that all AI systems analyze the same images.

To create the cohort dataset that reflects the real-world situation, all consecutive screening examinations, regardless of cancer status, between 1st January 2017 and 31st December 2017 were included. The RIS data was used to identify all screening examinations carried out across the three participating regions in 2017. The personal ID of the included screening participants was then linked to the NKBC data to identify all participants diagnosed with cancer within 36 months of screening (defined as cancer cases). This cohort dataset was used to determine the real ratio of participants diagnosed with cancer to the participants remaining healthy. This ratio of diagnosed to healthy was then used to ensure that the upscaling and bootstrapping methods of the case-control dataset represented the real-world setting (supplementary data).

### AI inferences and data quality control

In this study, prediction scores for the likelihood of cancer (inference results) from three AI systems from Lunit INSIGHT MMG (South Korea, v.1.1.7.2), Vara (Germany, v2.1) and Therapixel MammoScreen (France, v2.1.0) were used. All three AI systems make predictions on study level, meaning they analyze the four standard view images (LCC: Left craniocaudal, RCC: Right craniocaudal, LMLO: Left mediolateral oblique, and RMLO: Right mediolateral oblique) of one screening examination to produce a maximum left and right and overall score for the likelihood of cancer in an examination.

Screening mammograms for the participants included were transferred from the region’s radiology PACS system to a cloud platform service. The studies were processed by the three AI systems and the outputs were downloaded to our local storage server at Karolinska Institutet, as detailed by ref. ^15^. AI inferences, radiological assessment and NKBC data were linked and loaded in the R programming environment for quality control and downstream analysis^36^. For quality control, first we marked all missing values with NA to be correctly viewed as missing by the R programming environment. Next all variable names were checked for consistency and fixed if possible or removed otherwise. Variables included the assessment by first and second reader radiologist, imaging equipment information (manufacturer), examination year, participant age and breast density and cancer diagnosis. Exams that do not have inferences from all three AI systems or had missing radiologist decision information were removed. The maximum score for each AI system was assigned based on the aggregate left and right scores, with the highest score being assigned as the maximum score. The highest breast density category (A, B, C, D) was added based on the highest Lunit aggregated left and right density category. Participants diagnosed with cancer (based on the NKBC record) were then marked in the dataset and the time between the date for the screening exam and diagnosis was calculated. Next, the reference standard was set up to mark a screening examination as either a cancer case or healthy control. The reference standard was set up by marking exams from participants within 36 months before diagnosis as cancer cases, and all other exams (outside 36 months or never diagnosed with cancer) were marked as healthy controls. Finally, cancer diagnosis was marked as either in-situ, invasive or undefined. Finally, all screening examinations after May 2019 (April 2019-December 2021) were removed as we do not have 3-year follow-up information for these examinations. The data was then split into case-control and cohort datasets for downstream analysis.

### Evaluation outcome: overall AI performance

To evaluate overall AI performance for the various subsets and data selection methods, receiver operating characteristics (ROC) curves were drawn along with an estimation of the area under the curve (AUC). Subsequent assessment of differences in the AUC values of the models on different datasets, or subsets of the same data were assessed using the DeLong test^37^.

### Evaluation outcome: clinically relevant operating point

To evaluate and compare specific operating points of AI performance between different datasets and between AI and radiologists’ performance, the AI threshold to distinguish between cancer and healthy was calibrated based on the Cancer Detection Rate (CDR) of the radiologist double reader performance. Multiple accuracy outcome measures were then calculated, along with 95% confidence intervals (95%CI) to compare performance: the number of true positive (TP), false positive (FP), true negative (TN) and false negative (FN) assessments. The confusion matrix of these values was then used to calculate sensitivity, specificity, false positive rate (FPR), false negative rate (FNR), positive predictive value (PPV), abnormal interpretation rate (AIR) per 1000 exams and cancer detection rate (CDR) per 1000 exams. For all the validation errors described below we examined the CDR and FPR values as these are the most relevant factors in a clinical setting.

### Potential validation errors

Multiple potential validation errors were investigated in this work, a summary of the validation errors is given in Table 3.

**Table 3:** Summary of the validation error categories along with the suggested guideline and potential erroneous data selection methods

Validation error	Representative data	Non-representative data
1. Follow-up period and reference standard	3-year follow-up	1-year follow-up
		2-year follow-up
		4-year follow-up
3. Temporal selection	Balanced selection for each calendar year (2008–2019)	Early cases (2008–2012) and late controls (2015–2019)
		Early control (2008–2012) and late cases (2015–2019)
5. Population characteristics: Age	Representative age distribution (40–74)	Post-menopause cases (>55) and pre-menopause controls (≤55)
		Pre-menopause cases (≤55) and post-menopause controls (>55)
7. Population characteristics: Mammographic density	Representative mammographic density distribution	Density A and B
		Density C and D
		Density A, B and C
10. Cancer characteristics	Representative distribution	Invasive only
		In situ only
12. Image acquisition (equipment manufacturer)	GE data	Philips calibration
	Philips data	GE calibration
	Balanced multivendor data	GE case and Philips controls
		Philips case and GE control

### Follow-up period and reference standard

The reference standard, which marks mammography examinations as either diagnosed with cancer (cases) or healthy (controls), is used to assess the radiologists’ and AI performance. For the radiologists, we compare the outcome of the double reading to the reference standard. For AI the continuous score is compared to the reference standard to assess overall performance (ROC-AUC), or the continuous score is split to a binary decision at a specific threshold to calculate the AI accuracy statistics. When comparing the performance of the radiologists to the AI performance we need to determine the optimal reference standard where the two groups can be accurately compared, without any bias towards radiologists or AI. To determine the optimal reference standards, we adjusted the follow-up period used to mark examinations as either cancer or healthy and compared the radiologists and AI performance. The follow-up period was set to 12, 24, 36 or 48 months and the radiologist's sensitivity and specificity were compared to the AI performance on a ROC curve.

### Temporal selection

The VAI-B validation data contains a representative, 1:5, ratio of cancer cases and health controls for each calendar year represented in the dataset. To illustrate the importance of this representative selection we manipulated our dataset in two different ways and assessed the differences in AI performance on the manipulated data compared to the full VAI-B dataset. For the first manipulated dataset we choose cancer cases from early years (2008–2012) and merged this with healthy controls from later years (2015–2019). The second manipulated dataset consisted of early healthy controls (2008–2012) merged with late cancer cases (2015–2019).

### Population characteristics: age and mammographic density

To demonstrate the impact of screening participant age at examination on AI performance we subdivided the data to contain different age groups: 40–49, 50–69 and 70–74 years, and compared the AI performance on each group. To further demonstrate the importance of selecting the correct age distribution we created mixed case-control data sets containing cases from post-menopause participants (age >55 years) and controls from pre-menopause participants (age ≤55 years) and vice versa. The threshold of 55 years of age was chosen based on the most common menopausal age for woman. As some screening programs do not include women under 50 years old, this split makes the data more generalizable. Furthermore, as there is a correlation between age and mammographic density the distribution of both factors must reflect the screening population^16,17^. To demonstrate the impact of mammographic density we compared the AI performance of the four different mammographic density categories as assigned by the Lunit INSIGHT MMG (v.1.1.7.2) algorithm (A lowest mammographic density to D highest mammographic density). The inter-rater agreement for breast density between radiologists and the Lunit INSIGHT MMG model has previously been evaluated and found to have a fair level of agreement (matched rate of 68.2%)^38^. Different combinations of mammographic density categories (A and B, C and D, A and B and C) were also compared to each other to demonstrate the impact of not having a mammographic density distribution reflecting the screening population.

### Cancer characteristics

Breast cancer can be divided into two main categories, ductal carcinoma in situ, which may progress to invasive breast cancer^39^. To assess the AI system performance on these cancer categories we split the data to contain only invasive or in situ and compared the AUC values to the overall dataset containing both. To determine the impact of using data containing only one of these cancer types to calibrate AI systems we calibrated the AI system on only in situ or invasive cancers and then applied this threshold to the overall dataset.

### Image acquisition equipment

To demonstrate the importance of having validated on the correct mammography imaging equipment, we assessed and compared the AI performance on different imaging equipment. To demonstrate a potential validation error, we calibrated the AI systems on data from one imaging equipment manufacturer and then applied this threshold to data from a different equipment manufacturer. To further expand this validation error, we also mixed the cases and controls from different equipment manufacturers to show the extreme impact this could have specifically when calibrating the AI system for a specific setting. Finally, as the imaging equipment and image post-processing differ between the regions in Sweden from which the data was obtained, we compared the overall AI performance between the three regions (Östergötland, Västmanland, Södermanland).

### Statistical analysis and reporting metrics

As the case-control data is case enriched it does not reflect the real-world situation in terms of positive predictive value (PPV), false positive rate (FPR), false negative rate (FNR), cancer detection rate (CDR) and abnormal interpretation rate (AIR). For this reason, we need methods, upscaling, or bootstrapping, to adjust the results to reflect a real-world scenario. The weighting used for examinations of cancer cases and healthy controls was based on the ratio of cancer cases to healthy controls in the cohort dataset. Upscaling of the case-control data was done through duplication of healthy control exams to match the ratio of cancer cases and healthy controls in the cohort dataset. Bootstrapping was done through differential weighting between cases and controls, based on cancer incidence rate in the cohort data. To assess if these methods were viable for real-world applications, we used the cohort dataset as a reference and compared the upscaling and bootstrapping results to the cohort data. As the cohort data contains all examinations from 2017, we extracted all examinations from 2017 in the case-control dataset for this analysis. We then applied upscaling or weighted bootstrapping to this subset of the case-control data and compared the accuracy metrics for the radiologist double reading. Finally, we also demonstrate how using the case-control dataset as is (without bootstrapping or upscaling) produces results that are not reflective of real-world scenarios. In addition, we investigated the impact on the accuracy metrics when using case-control data with case-control ratio of 1:5 and 1:1, both through upscaling and bootstrapping.

## Supplementary information

Supplementary figures and tables

## Data Availability

Summary statistics of the AI systems output, and non-identifiable patient-level data, can be made available on request through the corresponding author. The medical images cannot be shared due to their nature as validation data, but establishing a research collaboration agreement would make it possible to submit AI models to process the images in this study and receive summary results.

## Code availability

The R (version 4.4.2) code used for the data quality control and statistical analysis of the data is available on GitHub (https://github.com/mammoai/Published-Scripts).
