# Supplementary material for: Simulating mismatch between calibration and target population in AI for mammography the retrospective VAIB study
Source: NPJ Digit Med. 2025 May 8;8:259. doi: 10.1038/s41746-025-01623-0 (PMC12062211; doi:10.1038/s41746-025-01623-0)
Supplement: Supplementary file 1 — Supplementary figures and tables [file 41746_2025_1623_MOESM1_ESM.pdf]

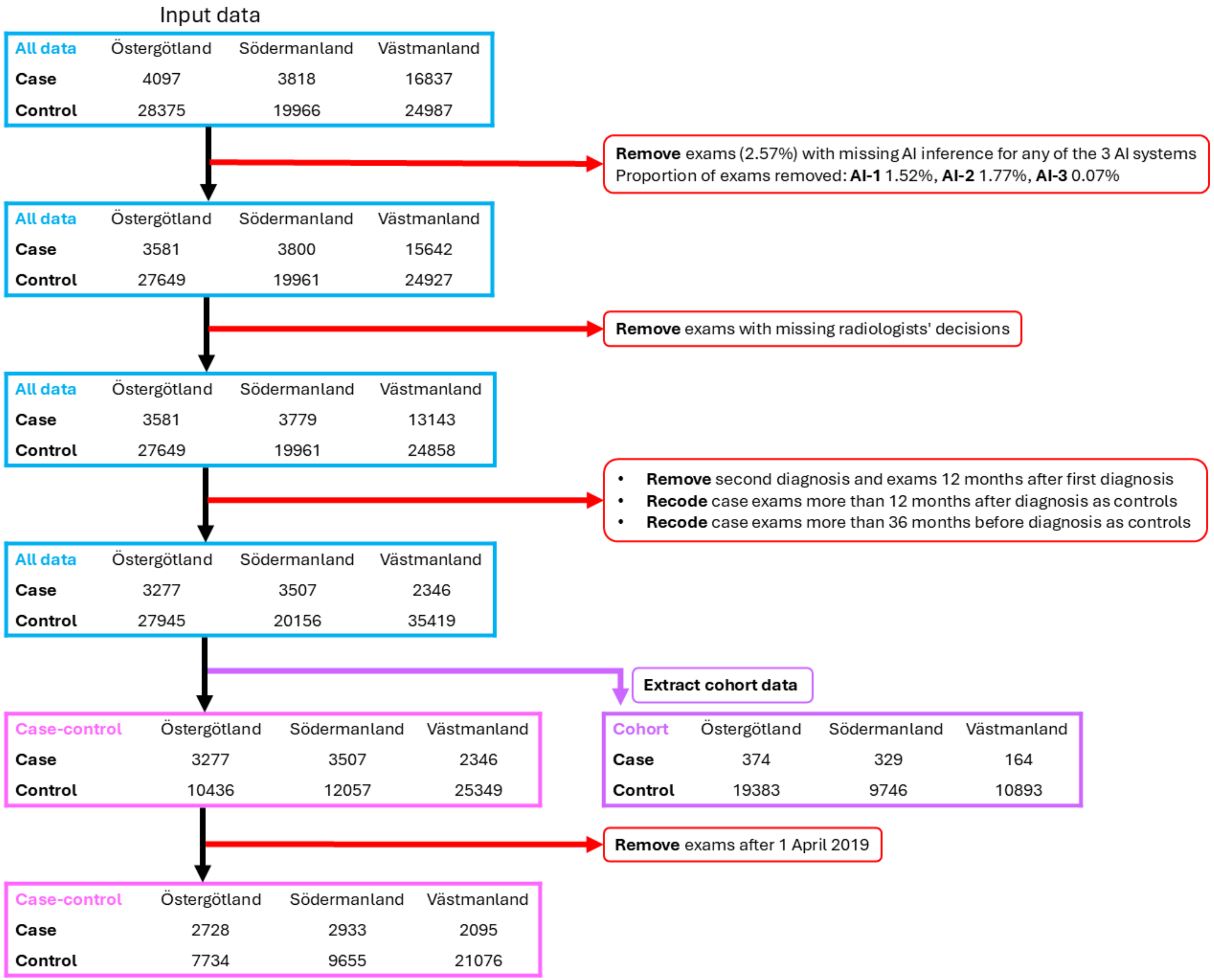

Supplementary Figure 1: Flow diagram of the data quality control procedure. *Outlining the steps from the initial input data to the final case-control and cohort datasets used in the analysis. Numbers indicate the number of mammography screening examinations.*

AI-1	1 year followup 1019862 (15340)	2 year followup 1027410 (7792)	3 year followup 1029780 (5422)	4 year followup 1031154 (4048)
1 year followup 1019862 (15340)	/	0.05 (14.6) ***	0.11 (32.3) ***	0.22 (70.0) ***
2 year followup 1027410 (7792)	-0.05 (-14.6) ***	/	0.06 (15.4) ***	0.17 (48.2) ***
3 year followup 1029780 (5422)	-0.11 (-32.3) ***	-0.06 (-15.4) ***	/	0.11 (31.1) ***
4 year followup 1031154 (4048)	-0.22 (-70.0) ***	-0.17 (-48.2) ***	-0.11 (-31.1) ***	/

P-value = *<0.05, **<0.01, *** <0.001
Rows and Columns: Categories with number of controls (cases).
Cells: Delta AUC (D-statistic)

AI-2	1 year followup 1019862 (15340)	2 year followup 1027410 (7792)	3 year followup 1029780 (5422)	4 year followup 1031154 (4048)
1 year followup 1019862 (15340)	/	0.05 (12.8) ***	0.11 (27.5) ***	0.22 (59.8) ***
2 year followup 1027410 (7792)	-0.05 (-12.8) ***	/	0.06 (13.8) ***	0.20 (42.6) ***
3 year followup 1029780 (5422)	-0.11 (-27.5) ***	-0.06 (-13.8) ***	/	0.11 (28.1) ***
4 year followup 1031154 (4048)	-0.22 (-59.8) ***	-0.20 (-42.6) ***	-0.11 (-28.1) ***	/

P-value = *<0.05, **<0.01, *** <0.001
Rows and Columns: Categories with number of controls (cases).
Cells: Delta AUC (D-statistic)

AI-3	1 year followup 1019862 (15340)	2 year followup 1027410 (7792)	3 year followup 1029780 (5422)	4 year followup 1031154 (4048)
1 year followup 1019862 (15340)	/	0.06 (14.4) ***	0.11 (29.5) ***	0.22 (64.1) ***
2 year followup 1027410 (7792)	-0.06 (-14.4) ***	/	0.05 (13.8) ***	0.16 (43.1) ***
3 year followup 1029780 (5422)	-0.11 (-29.5) ***	-0.05 (-13.8) ***	/	0.11 (28.3) ***
4 year followup 1031154 (4048)	-0.22 (-64.1) ***	-0.16 (-43.1) ***	-0.11 (-28.3) ***	/

P-value = *<0.05, **<0.01, *** <0.001
Rows and Columns: Categories with number of controls (cases).
Cells: Delta AUC (D-statistic)

Legend

Delta AUC	
(0.10 , ∞)	magenta
(0.05 , 0.10]	purple
(0.01 , 0.05]	blue
[0.01 , -0-01]	orange
(-0.01 , -0.05]	blue
(-0.05 , -0.10]	purple
(-0.10 , ∞)	magenta

( ) denotes excluded end points and [ ] to denote included end points

Supplementary Figure 2: Follow-up period and reference standard – AUROC comparison. *DeLong analysis to compare the AUC values for the different follow-up periods across the three AI systems.*

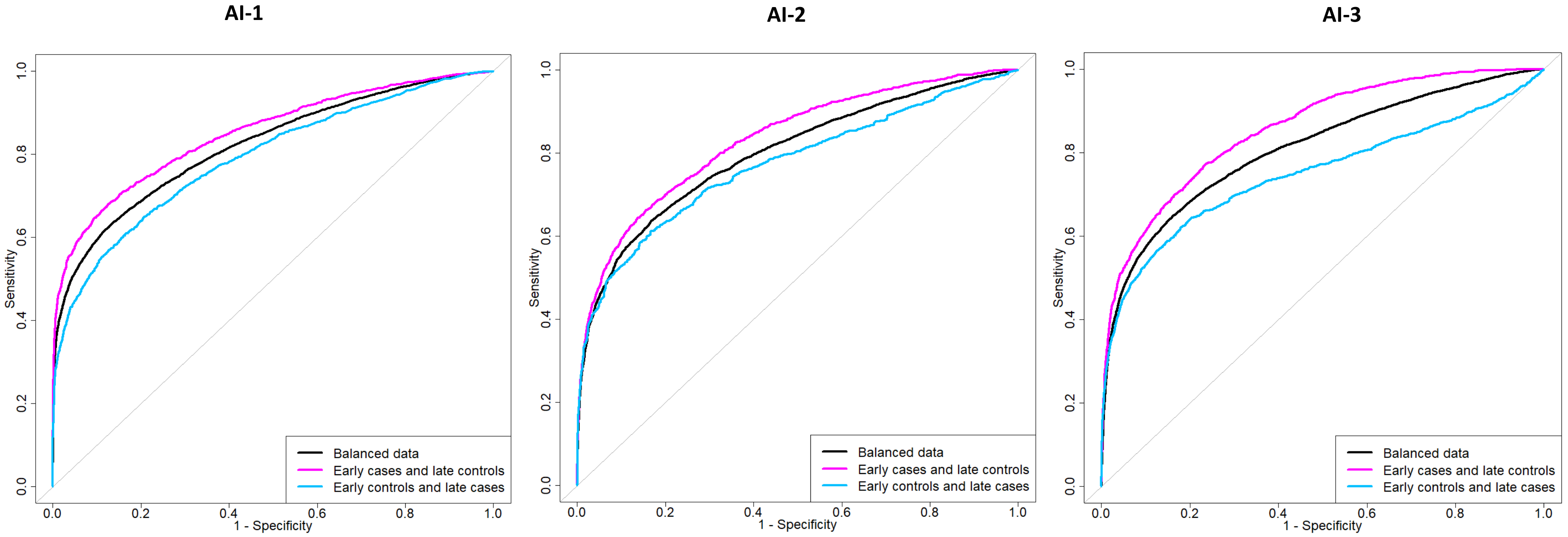

Supplementary Figure 3: ROC of Temporal selection. *ROC curve to demonstrate the impact of non-representative case-control data selection for each exam year for the three different AI systems. Selecting cases and controls from different time periods can inflate (early cases and late controls) or deflate (early controls and late cases) AI performance compared to the representative data.*

AI-1	Balanced data 1027410 (7792)	Early cases and late controls 217609 (1534)	Early controls and late cases 277189 (1954)
Balanced data 1027410 (7792)	/	-0.03 (-4.37) ***	0.03 (4.51) ***
Early cases and late controls 217609 (1534)	0.03 (4.37) ***	/	0.06 (6.95) ***
Early controls and late cases 277189 (1954)	-0.03 (-4.51) ***	-0.06 (-6.95) ***	/

P-value = *<0.05, **<0.01, *** <0.001
Rows and Columns: Categories with number of controls (cases).
Cells: Delta AUC (D-statistic)

AI-2	Balanced data 1027410 (7792)	Early cases and late controls 217609 (1534)	Early controls and late cases 277189 (1954)
Balanced data 1027410 (7792)	/	-0.03 (-5.04) ***	0.03 (3.69) ***
Early cases and late controls 217609 (1534)	0.03 (5.04) ***	/	0.06 (6.78) ***
Early controls and late cases 277189 (1954)	-0.03 (-3.69) ***	-0.06 (-6.78) ***	/

P-value = *<0.05, **<0.01, *** <0.001
Rows and Columns: Categories with number of controls (cases).
Cells: Delta AUC (D-statistic)

AI-3	Balanced data 1027410 (7792)	Early cases and late controls 217609 (1534)	Early controls and late cases 277189 (1954)
Balanced data 1027410 (7792)	/	-0.05 (-8.25) ***	0.06 (7.75) ***
Early cases and late controls 217609 (1534)	0.05 (8.25) ***	/	0.11 (12.3) ***
Early controls and late cases 277189 (1954)	-0.06 (-7.75) ***	-0.11 (-12.3) ***	/

P-value = *<0.05, **<0.01, *** <0.001
Rows and Columns: Categories with number of controls (cases).
Cells: Delta AUC (D-statistic)

Legend	
Delta AUC	
(0.10 , ∞)	
(0.05 , 0.10]	
(0.01 , 0.05]	
[0.01 , -0-01]	
(-0.01 , -0.05]	
(-0.05 , -0.10]	
(-0.10 , ∞)	

( ) denotes excluded end points and [ ] to denote included end points

Supplementary Figure 4: Temporal selection – AUROC comparison. *DeLong analysis to compare the AUC values for the different temporal data selection methods to indicate impact of representative versus non-representative (early and late case-control) selection.*

Supplementary Table 1: Temporal selection. *Accuracy metrics to demonstrate the impact of using non-representative data to calibrate the AI threshold and applying this threshold to the representative dataset, compared to calibrating the representative dataset to double radiologists cancer detection rate.*

AI system	Accuracy Metrics	Representative data calibration (95%CI)	Applying the Early cases and late controls data threshold		Applying the late cases and early controls data threshold	
			Early cases and late controls (95%CI)	Representative data (95%CI)	Late cases and early controls (95%CI)	Representative data (95%CI)
AI-1	True positives	4227	895	4114	1140	5039
	True negatives	959843	206238	968867	237081	871985
	False positives	67567	11371	58543	40108	155425
	False negatives	3565	639	3678	814	2753
	Sensitivity	0.54 (0.53-0.55)	0.58 (0.56-0.61)	0.53 (0.57-0.62)	0.58 (0.56-0.61)	0.65 (0.64-0.66)
	Specificity	0.93 (0.92-0.94)	0.95 (0.93-0.96)	0.94 (0.96-0.98)	0.86 (0.84-0.88)	0.85 (0.85-0.85)
	Pos Pred Value	0.06 (0.05-0.06)	0.07 (0.07-0.08)	0.07 (0.06-0.07)	0.03 (0.03-0.03)	0.03 (0.03-0.03)
	False Positive Rate	65.27 (64.77-65.73)	51.89 (50.97-52.67)	56.55 (56.10-57.01)	143.68 (142.13-144.96)	150.14 (149.43-150.83)
	False Negative Rate	3.44 (3.34-3.57)	2.92 (2.69-3.08)	3.55 (3.43-3.72)	2.92 (2.71-3.14)	2.66 (2.59-2.75)
	Cancer Detection Rate	4.08 (3.93-4.23)	4.08 (3.80-4.30)	3.97 (3.84-4.10)	4.08 (3.80-4.34)	4.87 (4.73-5.02)
	Abnormal interpretation rate	69.35 (69.00-69.97)	55.97 (54.98-57.06)	60.50 (59.97-61.00)	147.76 (146.47-148.81)	155.01 (154.32-155.69)
AI-2	True positives	4227	895	4272	1140	4959
	True negatives	935130	196610	929694	237878	854908
	False positives	92280	20999	97716	39311	172502
	False negatives	3565	639	3520	814	2833
	Sensitivity	0.54 (0.53-0.55)	0.58 (0.56-0.61)	0.55 (0.56-0.60)	0.58 (0.56-0.60)	0.64 (0.62-0.65)
	Specificity	0.91 (0.90-0.91)	0.90 (0.89-0.92)	0.91 (0.91-0.93)	0.86 (0.84-0.87)	0.83 (0.83-0.83)
	Pos Pred Value	0.04 (0.04-0.05)	0.04 (0.04-0.04)	0.04 (0.04-0.04)	0.03 (0.03-0.03)	0.03 (0.03-0.03)
	False Positive Rate	89.14 (88.59-89.75)	95.82 (94.60-96.94)	94.39 (93.81-95.05)	140.83 (139.62-142.08)	166.64 (166.00-167.32)
	False Negative Rate	3.44 (3.32-3.56)	2.92 (2.70-3.15)	3.40 (3.25-3.52)	2.92 (2.76-3.13)	2.74 (2.64-2.88)
	Cancer Detection Rate	4.08 (3.96-4.20)	4.08 (3.86-4.39)	4.13 (4.00-4.27)	4.08 (3.83-4.34)	4.79 (4.66-4.94)
	Abnormal interpretation rate	93.19 (92.65-93.84)	99.94 (98.72-100.98)	98.50 (97.94-99.09)	144.88 (143.58-146.36)	171.43 (170.59-172.21)
AI-3	True positives	4227	895	4220	1140	5039
	True negatives	945942	199864	946157	237145	862856
	False positives	81468	17745	81253	40044	164554
	False negatives	3565	639	3572	814	2753
	Sensitivity	0.54 (0.53-0.55)	0.58 (0.56-0.60)	0.54 (0.56-0.60)	0.58 (0.56-0.61)	0.65 (0.64-0.66)
	Specificity	0.92 (0.91-0.93)	0.92 (0.90-0.93)	0.92 (0.92-0.95)	0.86 (0.83-0.88)	0.84 (0.84-0.84)
	Pos Pred Value	0.05 (0.05-0.05)	0.05 (0.04-0.05)	0.05 (0.05-0.05)	0.03 (0.03-0.03)	0.03 (0.03-0.03)
	False Positive Rate	78.70 (78.12-79.27)	80.97 (79.95-82.12)	78.49 (77.85-79.16)	143.45 (142.25-144.64)	158.96 (158.12-159.70)
	False Negative Rate	3.44 (3.30-3.56)	2.92 (2.71-3.18)	3.45 (3.30-3.55)	2.92 (2.73-3.09)	2.66 (2.54-2.77)
	Cancer Detection Rate	4.08 (3.99-4.24)	4.08 (3.83-4.34)	4.07 (3.96-4.22)	4.08 (3.87-4.28)	4.87 (4.74-5.01)
	Abnormal interpretation rate	82.79 (82.29-83.25)	85.15 (83.85-86.25)	82.53 (82.01-83.19)	147.51 (146.05-148.75)	163.81 (163.17-164.45)

Supplementary Table 2: *Summary of the distribution of imaging equipment manufacturer, manufacturer model and software versions for the two non-* representative *temporally selected datasets.*

Category	Subgroup	Cases from early years and controls from later years		Cases from later years and controls from earlier years	
		**Cases**	**Controls**	**Cases**	**Controls**
**Manufacturer**	GE	1092	5876	1157	7258
	Philips	440	3340	788	8
**Philips model**	MDM 1.5	440	3340	0	6
	L50	0	0	788	0
	L30	0	0	0	2
**GE model**	Senograph DS ADS_32.10	5	0	0	23
	Senograph DS ADS_43.10.1	3	0	0	61
	Senograph DS VERSION ADS_53.10.1	11	0	0	79
	Senographe Essential ADS_41.0	30	0	0	291
	Senographe Essential ADS_41.02	2	0	0	32
	Senographe Essential ADS_43.0	9	0	0	169
	Senographe Essential ADS_43.10.1	132	0	0	1181
	Senographe Essential VERSION ADS_53.10	338	0	0	1165
	Senographe Essential VERSION ADS_53.10.1	331	0	0	3292
	Senographe Essential VERSION ADS_53.10.1.1	188	0	0	832
	Senographe Essential VERSION ADS_53.30	41	0	0	128
	Senographe Essential VERSION ADS_53.40	2	532	70	5
	Senographe Essential VERSION ADS_54.11	0	1439	192	0
	Senographe Essential VERSION ADS_55.20	0	128	8	0
	Senographe Pristina	0	3777	887	0
**Software versions***	GE	10x	8x	8x	11x
	Philips	4x	15x	12x	4x

*X number of different software versions between cases and controls for the individual manufacturers

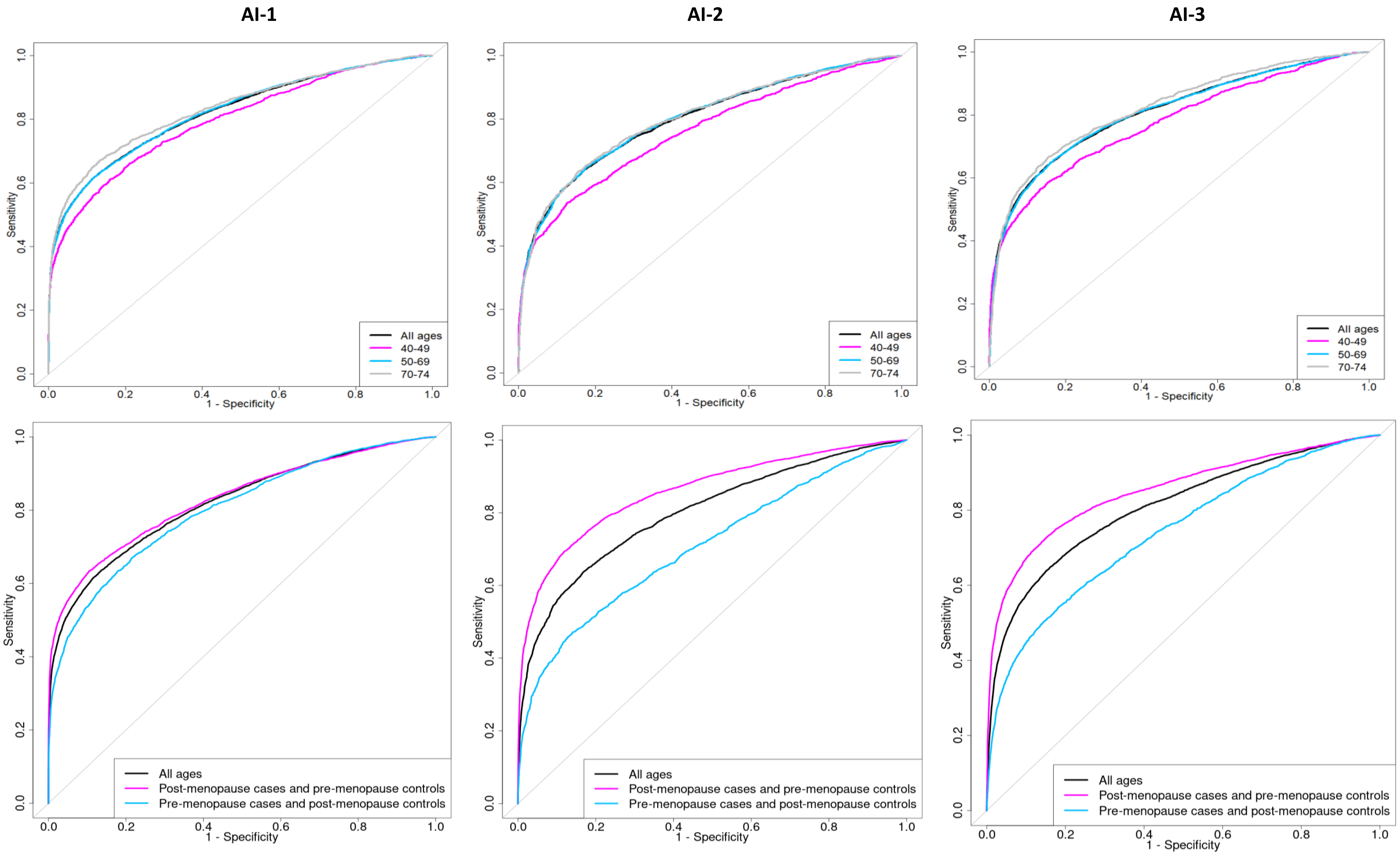

Supplementary Figure 5: Population characteristics: ROC of Age groups. *ROC curve to demonstrate the difference in AI performance for different age ranges **[top]** and for non- representative data selection of age groups between cases and controls compared to the representative dataset **[bottom]**. Selecting post-menopausal (age >55) cases and pre-menopausal (age <=55) controls inflates the AI performance, while selecting pre-menopausal cases and post-menopausal controls deflates the AI performance compared to the representative dataset.*

AI-1	All ages 1027410 (7792)	40-49 249696 (1257)	50-69 628038 (4888)	70-74 149623 (1647)
All ages 1027410 (7792)	/	0.02 (3.18) **	0.00 (-0.22)	-0.01 (-1.82)
40-49 249696 (1257)	-0.02 (-3.18) **	/	-0.02 (-3.19) **	-0.03 (-3.91) **
50-69 628038 (4888)	0.00 (-0.22)	0.02 (3.19) **	/	-0.01 (-1.59)
70-74 149623 (1647)	0.01 (1.82)	0.03 (3.91) **	0.01 (1.59)	/

P-value = *<0.05, **<0.01, *** <0.001
Rows and Columns: Categories with number of controls (cases).
Cells: Delta AUC (D-statistic)

AI-2	All ages 1027410 (7792)	40-49 249696 (1257)	50-69 628038 (4888)	70-74 149623 (1647)
All ages 1027410 (7792)	/	0.04 (4.44) ***	0.00 (-0.37)	0.00 (-0.47)
40-49 249696 (1257)	-0.04 (-4.44) ***	/	-0.04 (-4.49) ***	-0.04 (-4.02) **
50-69 628038 (4888)	0.00 (0.37)	0.04 (4.49) ***	/	0.00 (-0.22)
70-74 149623 (1647)	0.00 (0.47)	0.04 (4.02) **	0.00 (0.22)	/

P-value = *<0.05, **<0.01, *** <0.001
Rows and Columns: Categories with number of controls (cases).
Cells: Delta AUC (D-statistic)

AI-3	All ages 1027410 (7792)	40-49 249696 (1257)	50-69 628038 (4888)	70-74 149623 (1647)
All ages 1027410 (7792)	/	0.04 (4.13) ***	0.00 (0.16)	-0.01 (-1.9)
40-49 249696 (1257)	-0.04 (-4.13) ***	/	-0.04 (-3.9) ***	-0.05 (-4.78) **
50-69 628038 (4888)	0.00 (-0.16)	0.04 (3.9) ***	/	-0.01 (-1.9)
70-74 149623 (1647)	0.01 (1.9)	0.05 (4.78) **	-0.01 (1.9)	/

P-value = *<0.05, **<0.01, *** <0.001
Rows and Columns: Categories with number of controls (cases).
Cells: Delta AUC (D-statistic)

Legend

Delta AUC	
(0.10 , ∞)	
(0.05 , 0.10]	
(0.01 , 0.05]	
[0.01 , -0-01]	
(-0.01 , -0.05]	
(-0.05 , -0.10]	
(-0.10 , ∞)	

( ) denotes excluded end points and [ ] to denote included end points

Supplementary Figure 6**: **Population characteristics: Age groups – AUROC comparison *DeLong analysis to compare the AUC values for the different age categories compared to the* representative *dataset, which reflects the age range in the Swedish breast cancer screening program (40-74).*

AI-1	All ages 1027410 (7792)	Post-menopause cases and pre-menopause controls 784733 (5531)	Pre-menopause cases and post-menopause controls 320970 (2261)
All ages 1027410 (7792)	/	-0.01 (-2.06)	0.02 (3.22) **
Post-menopause cases and pre-menopause controls 784733 (5531)	0.01 (2.06)	/	0.03 (4.53) ***
Pre-menopause cases and post-menopause controls 320970 (2261)	-0.02 (-3.22) **	-0.03 (-4.53) ***	/

P-value = *<0.05, **<0.01, *** <0.001
Rows and Columns: Categories with number of controls (cases).
Cells: Delta AUC (D-statistic)

AI-2	All ages 1027410 (7792)	Post-menopause cases and pre-menopause controls 784733 (5531)	Pre-menopause cases and post-menopause controls 320970 (2261)
All ages 1027410 (7792)	/	-0.06 (-13.99) ***	0.09 (13.24) ***
Post-menopause cases and pre-menopause controls 784733 (5531)	0.06 (13.99) ***	/	0.15 (21.64) ***
Pre-menopause cases and post-menopause controls 320970 (2261)	0.09 (13.24) ***	-0.15 (-21.64) ***	/

P-value = *<0.05, **<0.01, *** <0.001
Rows and Columns: Categories with number of controls (cases).
Cells: Delta AUC (D-statistic)

AI-3	All ages 1027410 (7792)	Post-menopause cases and pre-menopause controls 784733 (5531)	Pre-menopause cases and post-menopause controls 320970 (2261)
All ages 1027410 (7792)	/	-0.04 (-9.86) ***	0.07 (10.47) ***
Post-menopause cases and pre-menopause controls 784733 (5531)	0.04 (9.86) ***	/	0.11 (16.66) ***
Pre-menopause cases and post-menopause controls 320970 (2261)	-0.07 (-10.47) ***	-0.11 (-16.66) ***	/

P-value = *<0.05, **<0.01, *** <0.001
Rows and Columns: Categories with number of controls (cases).
Cells: Delta AUC (D-statistic)

Legend	
Delta AUC	
(0.10 , ∞)	
(0.05 , 0.10]	
(0.01 , 0.05]	
[0.01 , -0-01]	
(-0.01 , -0.05]	
(-0.05 , -0.10]	
(-0.10 , ∞)	

( ) denotes excluded end points and
[ ] to denote included end points

***Supplementary Figure 7**: **Population characteristics: Age young and old – AUROC comparison.** DeLong analysis to compare the AUC values for the non-* representative *pre and post-menopause (split at age, 55, of menopause) data selection compared to the* representative *dataset, which reflects the age range in the Swedish breast cancer screening program (40-74).*

Supplementary Table 3: Population characteristics: Age. *Accuracy metrics to demonstrate the impact of using different age ranges to calibrate the AI threshold and then applying these thresholds to the representative dataset compared to calibrating the representative dataset to double radiologists' cancer detection rate.*

AI System	Accuracy Metrics	Representative data calibration (95%CI)	Applying the post-menopause calibration threshold		Applying the the pre-menopause calibration threshold		Applying the 40-49 calibration threshold		Applying the 50-69 calibration threshold		Applying the 70+ calibration threshold	
			Post-menopause data (95%CI)	Representative data (95%CI)	Pre-menopause data (95%CI)	Representative data (95%CI)	40-49 data (95%CI)	Representative data (95%CI)	50-69 data (95%CI)	Representative data (95%CI)	70+ data (95%CI)	Representative data (95%CI)
AI-1	True positives	4227	3227	4386	1319	4881	734	5035	2851	4533	961	4142
	True negatives	959843	718870	947109	282681	895706	153974	872359	629814	934883	217402	967396
	False positives	67567	65849	80301	38179	131704	24446	155051	63644	92527	16355	60014
	False negatives	3565	2304	3406	942	2911	523	2757	2037	3259	686	3650
	Sensitivity	0.54 (0.53-0.55)	0.58 (0.57-0.59)	0.56 (0.56-0.590)	0.58 (0.56-0.60)	0.63 (0.58-0.62)	0.58 (0.55-0.61)	0.65 (0.57-0.63)	0.58 (0.57-0.59)	0.58 (0.57-0.60)	0.58 (0.56-0.61)	0.53 (0.54-0.59)
	Specificity	0.93 (0.92-0.94)	0.92 (0.91-0.93)	0.92 (0.92-0.94)	0.88 (0.86-0.90)	0.87 (0.82-0.86)	0.86 (0.84-0.89)	0.85 (0.78-0.82)	0.91 (0.90-0.92)	0.91 (0.90-0.92)	0.93 (0.91-0.95)	0.94 (0.95-0.97)
	Pos Pred Value	0.06 (0.05-0.06)	0.05 (0.05-0.05)	0.05 (0.05-0.06)	0.03 (0.03-0.04)	0.04 (0.04-0.04)	0.03 (0.03-0.03)	0.03 (0.03-0.03)	0.04 (0.04-0.05)	0.05 (0.05-0.05)	0.06 (0.05-0.06)	0.07 (0.07-0.07)
	False Positive Rate	65.27 (64.86-65.74)	83.33 (82.63-83.83)	77.57 (76.97-78.00)	118.16 (116.66-119.10)	127.23 (126.56-127.91)	136.06 (133.93-137.60)	149.78 (149.04-150.39)	91.14 (90.36-91.84)	89.38 (88.84-89.82)	69.48 (68.50-70.44)	57.97 (57.48-58.45)
	False Negative Rate	3.44 (3.34-3.55)	2.92 (2.78-3.06)	3.29 (3.20-3.41)	2.92 (2.74-3.13)	2.81 (2.71-2.92)	2.91 (2.68-3.14)	2.66 (2.55-2.74)	2.92 (2.80-3.08)	3.15 (3.02-3.27)	2.91 (2.69-3.17)	3.53 (3.41-3.63)
	Cancer Detection Rate	4.08 (3.94-4.21)	4.08 (3.91-4.21)	4.24 (4.11-4.37)	4.08 (3.83-4.30)	4.72 (4.59-4.89)	4.09 (3.76-4.35)	4.86 (4.75-5.03)	4.08 (3.94-4.24)	4.38 (4.25-4.52)	4.08 (3.83-4.32)	4.00 (3.88-4.13)
	Abnormal interpretation rate	69.41 (68.78-69.94)	87.42 (86.82-87.99)	81.85 (81.32-82.30)	122.17 (121.19-123.32)	131.91 (131.13-132.65)	140.14 (138.49-142.01)	154.67 (153.97-155.41)	95.23 (94.55-95.96)	93.73 (93.17-94.20)	73.59 (72.51-74.47)	61.96 (61.52-62.51)
AI-2	True positives	4227	3227	4386	1319	4881	734	5756	2851	4483	961	3861
	True negatives	945942	706546	947109	282681	895706	145005	721531	610999	911242	206069	956164
	False positives	81468	78173	80301	38179	131704	33415	305879	82459	116168	27688	71246
	False negatives	3565	2304	3406	942	2911	523	2036	2037	3309	686	3931
	Sensitivity	0.54 (0.53-0.55)	0.58 (0.57-0.59)	0.56 (0.56-0.59)	0.58 (0.56-0.60)	0.63 (0.58-0.62)	0.58 (0.56-0.61)	0.74 (0.65-0.69)	0.58 (0.57-0.59)	0.58 (0.56-0.59)	0.58 (0.56-0.61)	0.50 (0.49-0.53)
	Specificity	0.92 (0.91-0.93)	0.90 (0.89-0.91)	0.92 (0.92-0.94)	0.88 (0.86-0.90)	0.87 (0.82-0.86)	0.81 (0.78-0.85)	0.70 (0.57-0.74)	0.88 (0.87-0.89)	0.89 (0.88-0.90)	0.88 (0.86-0.90)	0.93 (0.93-0.95)
	Pos Pred Value	0.05 (0.05-0.05)	0.04 (0.04-0.04)	0.05 (0.05-0.05)	0.03 (0.03-0.04)	0.04 (0.04-0.04)	0.02 (0.02-0.02)	0.02 (0.02-0.02)	0.03 (0.03-0.03)	0.04 (0.04-0.04)	0.03 (0.03-0.04)	0.05 (0.05-0.05)
	False Positive Rate	78.70 (78.17-79.23)	98.92 (98.34-99.58)	77.57 (76.97-78.00)	118.16 (116.66-119.10)	127.23 (126.56-127.91)	185.97 (184.19-187.77)	295.48 (294.59-296.31)	118.08 (117.47-118.99)	112.22 (111.60-112.70)	117.62 (116.46-119.15)	68.82 (68.33-69.43)
	False Negative Rate	3.44 (3.33-3.57)	2.92 (2.79-3.02)	3.29 (3.19-3.41)	2.92 (2.74-3.13)	2.81 (2.71-2.92)	2.91 (2.69-3.17)	1.97 (1.87-2.04)	2.92 (2.77-3.07)	3.20 (3.09-3.32)	2.91 (2.65-3.18)	3.80 (3.68-3.92)
	Cancer Detection Rate	4.08 (3.95-4.20)	4.08 (3.92-4.23)	4.24 (4.11-4.37)	4.08 (3.83-4.30)	4.71 (4.58-4.89)	4.09 (3.80-4.39)	5.56 (5.37-5.69)	4.08 (3.89-4.24)	4.33 (4.18-4.47)	4.08 (3.79-4.34)	3.73 (3.64-3.83)
	Abnormal interpretation rate	82.77 (82.15-83.33)	103.03 (102.36-103.67)	81.85 (81.32-82.31)	122.17 (121.19-123.32)	131.91 (131.13-132.65)	190.09 (188.27-191.57)	301.01 (300.21-302.17)	122.16 (121.40-122.85)	116.60 (115.89-117.08)	121.69 (120.62-122.82)	72.52 (72.14-73.03)
AI-3	True positives	4227	3227	4235	1319	5279	734	5564	2851	4509	961	3878
	True negatives	945942	706546	945702	275708	828424	151133	782363	617766	921808	211808	966999
	False positives	81468	78173	81708	45152	198986	27287	245047	75693	105602	21949	60411
	False negatives	3565	2304	3557	942	2513	523	2228	2037	3283	686	3914
	Sensitivity	0.54 (0.53-0.55)	0.58 (0.57-0.59)	0.54 (0.53-0.55)	0.58 (0.56-0.60)	0.68 (0.61-0.66)	0.58 (0.56-0.61)	0.71 (0.64-0.69)	0.58 (0.57-0.59)	0.58 (0.56-0.59)	0.58 (0.56-0.60)	0.50 (0.49-0.54)
	Specificity	0.92 (0.91-0.93)	0.90 (0.89-0.91)	0.92 (0.91-0.93)	0.86 (0.84-0.87)	0.81 (0.73-0.87)	0.85 (0.81-0.87)	0.76 (0.63-0.70)	0.89 (0.88-0.90)	0.90 (0.89-0.91)	0.91 (0.90-0.92)	0.94 (0.94-0.95)
	Pos Pred Value	0.05 (0.05-0.05)	0.04 (0.04-0.04)	0.05 (0.05-0.05)	0.03 (0.03-0.03)	0.03 (0.03-0.03)	0.03 (0.03-0.03)	0.02 (0.02-0.02)	0.04 (0.04-0.04)	0.04 (0.04-0.04)	0.04 (0.04-0.04)	0.06 (0.06-0.06)
	False Positive Rate	78.70 (78.17-79.23)	98.92 (98.33-99.58)	78.93 (78.21-79.53)	139.74 (138.64-141.11)	192.22 (191.55-192.99)	151.87 (149.95-153.84)	236.71 (235.90-237.63)	108.40 (107.73-109.06)	102.01 (101.43-102.55)	93.24 (92.24-94.52)	58.36 (57.91-58.90)
	False Negative Rate	3.44 (3.33-3.57)	2.93 (2.79-3.03)	3.44 (3.34-3.53)	2.92 (2.77-3.07)	2.43 (2.33-2.53)	2.91 (2.58-3.15)	2.15 (2.05-2.27)	2.92 (2.81-3.03)	3.17 (3.07-3.32)	2.91 (2.66-3.12)	3.78 (3.65-3.90)
	Cancer Detection Rate	4.08 (3.95-4.20)	4.08 (3.93-4.23)	4.09 (3.95-4.25)	4.08 (3.85-4.27)	5.10 (4.91-5.27)	4.09 (3.80-4.46)	5.38 (5.23-5.49)	4.08 (3.96-4.22)	4.36 (4.25-4.50)	4.08 (3.80-4.31)	3.75 (3.65-3.86)
	Abnormal interpretation rate	82.77 (82.15-83.33)	103.03 (102.36-103.67)	82.99 (82.46-83.48)	143.89 (142.41-144.99)	197.26 (196.51-198.39)	155.97 (154.19-157.44)	242.05 (241.01-243.00)	112.47 (111.57-113.37)	106.38 (105.70-106.99)	97.41 (95.99-98.39)	62.16 (61.59-62.54)

Pre-menopause is defined as screening participants less than or equal to 55 years of age.

Post-menopause is defined as screening participants more than 55 years of age.

Supplementary Table 4: Population characteristics: Age. *Accuracy metrics to demonstrate the impact of using non- representative age data to calibrate the AI threshold and then applying these thresholds to the representative dataset compared to calibrating the representative dataset to double radiologists' cancer detection rate.*

AI Systems	Accuracy Metrics	Representative data calibration (95%CI)	Applying the post-menopause cases and pre-menopause controls threshold		Applying the pre-menopause cases and post-menopause controls threshold	
			Post-menopause cases pre-menopause controls data (95%CI)	Representative data (95%CI)	Pre-menopause cases post-menopause controls data (95%CI)	Representative data (95%CI)
AI-1	True positives	4227	3227	4386	1319	4882
	True negatives	959843	730111	947109	277870	895626
	False positives	67567	54622	80301	43100	131784
	False negatives	3565	2304	3406	942	2910
	Sensitivity	0.54 (0.53-0.55)	0.58 (0.57-0.60)	0.56 (0.58-0.61)	0.58 (0.57-0.60)	0.62 (0.56-0.60)
	Specificity	0.93 (0.93-0.94)	0.93 (0.92-0.94)	0.92 (0.94-0.95)	0.87 (0.85-0.88)	0.87 (0.80-0.84)
	Pos Pred Value	0.06 (0.06-0.06)	0.06 (0.05-0.06)	0.05 (0.05-0.05)	0.03 (0.03-0.03)	0.04 (0.04-0.04)
	False Positive Rate	65.27 (64.86-65.74)	69.12 (68.41-69.58)	77.57 (77.16-78.05)	133.34 (132.06-134.52)	127.30 (126.68-128.12)
	False Negative Rate	3.44 (3.34-3.55)	2.92 (2.76-3.03)	3.29 (3.20-3.44)	2.91 (2.77-3.10)	2.81 (2.70-2.93)
	Cancer Detection Rate	4.08 (3.94-4.21)	4.08 (3.93-4.23)	4.28 (4.09-4.36)	4.08 (3.86-4.31)	4.72 (4.61-4.84)
	Abnormal interpretation rate	69.41 (68.78-69.94)	73.23 (72.56-73.78)	81.79 (81.33-82.37)	137.38 (136.23-138.79)	132.02 (131.21-132.58)
AI-2	True positives	4227	3227	4220	1320	5361
	True negatives	945942	743870	935423	230947	788244
	False positives	81468	40863	91987	90023	239166
	False negatives	3565	2304	3572	941	2431
	Sensitivity	0.54 (0.53-0.55)	0.58 (0.57-0.59)	0.54 (0.64-0.66)	0.58 (0.56-0.60)	0.69 (0.53-0.57)
	Specificity	0.92 (0.91-0.93)	0.95 (0.94-0.95)	0.91 (0.96-0.96)	0.72 (0.69-0.74)	0.77 (0.55-0.59)
	Pos Pred Value	0.05 (0.05-0.05)	0.07 (0.07-0.08)	0.04 (0.04-0.05)	0.01 (0.01-0.01)	0.02 (0.02-0.02)
	False Positive Rate	78.70 (78.17-79.23)	51.71 (51.23-52.26)	88.86 (88.26-89.50)	278.51 (276.63-279.90)	231.03 (230.29-231.97)
	False Negative Rate	3.44 (3.33-3.60)	2.92 (2.81-3.03)	3.45 (3.34-3.56)	2.91 (2.65-3.13)	2.35 (2.27-2.45)
	Cancer Detection Rate	4.08 (3.95-4.20)	4.08 (3.95-4.25)	4.08 (3.98-4.19)	4.08 (3.86-4.31)	5.18 (5.04-5.34)
	Abnormal interpretation rate	82.77 (82.15-83.33)	55.80 (55.25-56.30)	92.97 (92.39-93.46)	282.52 (280.82-284.06)	236.22 (235.54-237.06)
AI-3	True positives	3711	3231	4241	791	3428
	True negatives	976029	390213	945061	585746	987466
	False positives	51381	20558	82349	30840	39944
	False negatives	4081	2300	3551	1470	4364
	Sensitivity	0.48 (0.47-0.49)	0.58 (0.57-0.60)	0.54 (0.62-0.65)	0.35 (0.33-0.37)	0.44 (0.30-0.45)
	Specificity	0.95 (0.95-0.95)	0.95 (0.94-0.95)	0.92 (0.96-0.97)	0.95 (0.94-0.96)	0.96 (0.89-0.98)
	Pos Pred Value	0.07 (0.07-0.07)	0.14 (0.13-0.14)	0.05 (0.05-0.05)	0.03 (0.03-0.03)	0.08 (0.08-0.08)
	False Positive Rate	49.63 (49.28-50.20)	49.38 (48.77-49.93)	79.55 (79.00-80.20)	49.84 (49.28-50.48)	38.59 (38.15-38.91)
	False Negative Rate	3.94 (3.85-4.10)	5.53 (5.28-5.76)	3.43 (3.32-3.54)	2.38 (2.25-2.49)	4.22 (4.04-4.33)
	Cancer Detection Rate	3.59 (3.46-3.71)	7.76 (7.50-7.96)	4.10 (3.99-4.25)	1.28 (1.20-1.37)	3.31 (3.21-3.41)
	Abnormal interpretation rate	53.18 (52.77-53.64)	57.19 (56.43-57.82)	83.65 (83.02-84.11)	51.12 (50.51-51.79)	41.90 (41.61-42.30)

Pre-menopause is defined as screening participants less than or equal to 55 years of age.
Post-menopause is defined as screening participants more than 55 years of age.

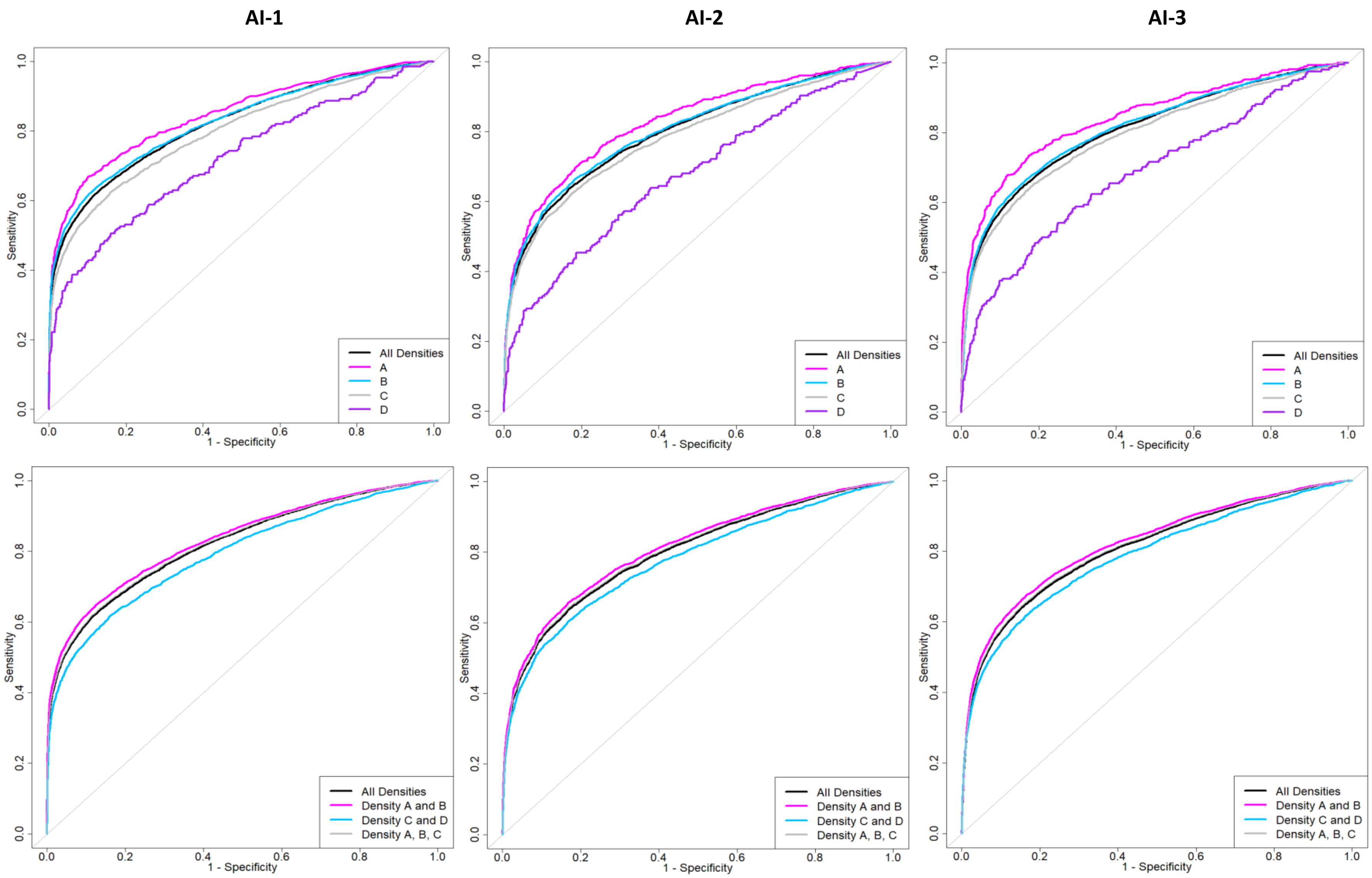

Supplementary Figure 8: Population characteristics: ROC of Mammographic density. *ROC curve to show the performance of AI systems on different mammographic breast density categories (A least dense to D most dense)* ***[Top].*** *The performance of the AI system for data containing only low density (category A and B), high density (C and D), and all density categories except for density D (grey line is overlapped by the black "All Densities" line) compared to the representative dataset* ***[bottom]****. The density categories were obtained from the Lunit AI system.*

AI-1	Density A 146124 (677)	Density B 548723 (4187)	Density C 298968 (2734)	Density D 33568 (194)
Density A 146124 (677)	/	0.02 (2.29)	0.05 (4.9) ***	0.13 (5.47) ***
Density B 548723 (4187)	-0.02 (-2.29)	/	0.03 (4.41) ***	0.11 (4.8) ***
Density C 298968 (2734)	-0.05 (-4.9) ***	-0.03 (-4.41) ***	/	0.08 (3.46) **
Density D 33568 (194)	-0.13 (-5.47) ***	-0.11 (-4.8) ***	-0.08 (-3.46) **	/

P-value = *<0.05, **<0.01, *** <0.001
Rows and Columns: Categories with number of controls (cases).
Cells: Delta AUC (D-statistic)

AI-2	Density A 146124 (677)	Density B 548723 (4187)	Density C 298968 (2734)	Density D 33568 (194)
Density A 146124 (677)	/	0.03 (2.46)	0.05 (4.28) ***	0.15 (6.61) ***
Density B 548723 (4187)	-0.03 (-2.46)	/	0.02 (3.12) *	0.12 (5.94) ***
Density C 298968 (2734)	-0.05 (-4.28) ***	-0.02 (-3.12) *	/	0.10 (4.94) ***
Density D 33568 (194)	-0.15 (-6.61) ***	-0.12 (-5.94) ***	-0.10 (-4.94) ***	/

P-value = *<0.05, **<0.01, *** <0.001
Rows and Columns: Categories with number of controls (cases).
Cells: Delta AUC (D-statistic)

AI-3	Density A 146124 (677)	Density B 548723 (4187)	Density C 298968 (2734)	Density D 33568 (194)
Density A 146124 (677)	/	0.03 (2.98)	0.05 (4.73) ***	0.15 (6.76) ***
Density B 548723 (4187)	-0.03 (-2.98)	/	0.02 (3.08) *	0.12 (5.88) ***
Density C 298968 (2734)	-0.05 (-4.73) ***	-0.02 (-3.08) *	/	0.10 (4.92) ***
Density D 33568 (194)	-0.15 (-6.76) ***	-0.12 (-5.88) ***	-0.10 (-4.92) ***	/

P-value = *<0.05, **<0.01, *** <0.001
Rows and Columns: Categories with number of controls (cases).
Cells: Delta AUC (D-statistic)

Legend	
Delta AUC	
(0.10 , ∞)	(pink)
(0.05 , 0.10]	(purple)
(0.01 , 0.05]	(blue)
[0.01 , -0-01]	(orange)
(-0.01 , -0.05]	(blue)
(-0.05 , -0.10]	(purple)
(-0.10 , ∞)	(pink)

( ) denotes excluded end points and [ ] to denote included end points

***Supplementary Figure 9: Population characteristics: Mammographic density categories* – AUROC comparison.** *DeLong analysis to compare the AUC values for the different mammographic breast density categories (A, B, C, D), where density A is the lowest and density D is the highest density. The density categories were obtained from the Lunit AI system.*

AI-1	All Densities 1027410 (7792)	Density A and B 690109 (4864)	Density C and D 415465 (2928)	Density A, B, C 1077943 (7598)
All Densities 1027410 (7792)	/	-0.01 (-2.54)	0.03 (5.11) ***	0.00 (-0.64)
Density A and B 690109 (4864)	0.01 (2.54)	/	0.04 (6.69) ***	0.01 (1.97)
Density C and D 415465 (2928)	-0.03 (-5.11) ***	-0.04 (-6.69) ***	/	-0.03 (-5.55) ***
Density A, B, C 1077943 (7598)	0.00 (-0.64)	-0.01 (-1.97)	0.03 (-5.55) ***	/

P-value = *<0.05, **<0.01, *** <0.001
Rows and Columns: Categories with number of controls (cases).
Cells: Delta AUC (D-statistic)

AI-2	All Densities 1027410 (7792)	Density A and B 690109 (4864)	Density C and D 415465 (2928)	Density A, B, C 1077943 (7598)
All Densities 1027410 (7792)	/	-0.01 (-2.51)	0.02 (3.87) ***	0.00 (-0.81)
Density A and B 690109 (4864)	0.01 (2.51)	/	0.03 (5.51) ***	0.01 (1.78)
Density C and D 415465 (2928)	-0.02 (-3.87) ***	-0.03 (-5.51) ***	/	-0.02 (-4.43) ***
Density A, B, C 1077943 (7598)	0.00 (-0.81)	-0.01 (-1.78)	-0.02 (-4.43) ***	/

P-value = *<0.05, **<0.01, *** <0.001
Rows and Columns: Categories with number of controls (cases).
Cells: Delta AUC (D-statistic)

AI-3	All Densities 1027410 (7792)	Density A and B 690109 (4864)	Density C and D 415465 (2928)	Density A, B, C 1077943 (7598)
All Densities 1027410 (7792)	/	-0.01 (-2.73) *	0.02 (3.78) ***	0.00 (-0.74)
Density A and B 690109 (4864)	0.01 (2.73) *	/	0.03 (5.59) ***	0.01 (2.06)
Density C and D 415465 (2928)	-0.02 (-3.78) ***	-0.03 (-5.59) ***	/	-0.02 (-4.30) ***
Density A, B, C 1077943 (7598)	0.00 (-0.74)	-0.01 (-2.06)	-0.02 (-4.30) ***	/

P-value = *<0.05, **<0.01, *** <0.001
Rows and Columns: Categories with number of controls (cases).
Cells: Delta AUC (D-statistic)

Legend	
Delta AUC	
(0.10 , ∞)	
(0.05 , 0.10]	
(0.01 , 0.05]	
[0.01 , -0-01]	
(-0.01 , -0.05]	
(-0.05 , -0.10]	
(-0.10 , ∞)	

( ) denotes excluded end points and [ ] to denote included end points

Supplementary Figure 10: Population characteristics: Mammographic density groups – AUROC comparison. *DeLong analysis to compare the AUC values for the different density groupings, low density (category A and B), high density (category C and D) and all densities except for density category D compared to the representative dataset, which reflects the mammographic breast density category distribution in the Swedish breast cancer screening population.*

Supplementary Table 5: Population characteristics: Mammographic density. *Accuracy metrics to demonstrate the impact of using various combinations of density categories to calibrate the AI threshold and then applying these thresholds to the* representative *dataset compared to the calibrating the* representative *dataset to double radiologists cancer detection rate.*

AI System	Accuracy Metrics	Representative data calibration (95%CI)	Applying the Density A and B data threshold		Applying the Density C and D data threshold		Applying the DensityA, B and C data threshold	
			Density A and B data (95%CI)	Representative data (95%CI)	Density C and D data (95%CI)	Representative data (95%CI)	Density A,B and C data (95%CI)	Representative data (95%CI)
	True positives	4227	2838	4520	1708	4578	4432	4518
	True negatives	959843	639963	935682	360517	929866	983234	936080
	False positives	67567	50146	91728	54948	97544	94709	91330
	False negatives	3565	2026	3272	1220	3214	3166	3274
	Sensitivity	0.54 (0.53-0.55)	0.58 (0.57-0.59)	0.58 (0.57-0.62)	0.58 (0.56-0.60)	0.59 (0.55-0.60)	0.58 (0.57-0.59)	0.58 (0.57-0.59)
**AI-1**	Specificity	0.93 (0.93-0.94)	0.93 (0.92-0.94)	0.91 (0.92-0.94)	0.87 (0.85-0.88)	0.90 (0.85-0.91)	0.91 (0.90-0.92)	0.91 (0.91-0.92)
	Pos Pred Value	0.06 (0.06-0.06)	0.05 (0.05-0.06)	0.05 (0.05-0.05)	0.03 (0.03-0.03)	0.05 (0.05-0.05)	0.04 (0.04-0.05)	0.05 (0.05-0.05)
	False Positive Rate	65.27 (64.80-65.76)	72.16 (71.61-72.55)	88.61 (88.10-89.35)	131.33 (130.29-132.51)	94.23 (93.65-94.73)	87.25 (86.79-87.81)	88.22 (87.74-88.71)
	False Negative Rate	3.44 (3.33-3.57)	2.92 (2.76-3.06)	3.16 (3.04-3.25)	2.92 (2.74-3.10)	3.11 (3.00-3.20)	2.92 (2.80-3.00)	3.16 (3.01-3.27)
	Cancer Detection Rate	4.08 (3.98-4.24)	4.08 (3.94-4.24)	4.37 (4.22-4.50)	4.08 (3.83-4.25)	4.42 (4.30-4.56)	4.08 (3.96-4.21)	4.36 (4.24-4.48)
	Abnormal interpretation rate	69.38 (68.66-69.78)	76.24 (75.61-76.90)	93.01 (92.50-93.72)	135.41 (134.11-136.43)	98.64 (98.01-99.23)	91.40 (90.74-91.92)	92.53 (92.08-93.24)
	True positives	4227	2838	4368	1707	4823	4432	4490
	True negatives	935261	618165	922765	352065	868857	953761	910473
	False positives	92149	71944	104645	63400	158553	124182	116937
	False negatives	3565	2026	3424	1221	2969	3166	3302
	Sensitivity	0.54 (0.53-0.56)	0.583 (0.570-0.596)	0.56 (0.56-0.59)	0.58 (0.56-0.60)	0.62 (0.57-0.60)	0.58 (0.57-0.59)	0.58 (0.57-0.59)
**AI-2**	Specificity	0.91 (0.90-0.91)	0.896 (0.884-0.906)	0.90 (0.90-0.91)	0.85 (0.83-0.86)	0.85 (0.80-0.87)	0.89 (0.87-0.89)	0.89 (0.88-0.90)
	Pos Pred Value	0.04 (0.04-0.05)	0.038 (0.036-0.039)	0.04 (0.04-0.04)	0.03 (0.03-0.03)	0.03 (0.03-0.03)	0.03 (0.03-0.04)	0.04 (0.04-0.04)
	False Positive Rate	89.01 (88.42-89.59)	103.52 (102.79-104.24)	101.09 (100.56-101.77)	151.53 (150.46-152.51)	153.16 (152.34-153.85)	114.40 (113.80-115.02)	112.96 (112.18-113.68)
	False Negative Rate	3.44 (3.34-3.56)	2.92 (2.78-3.06)	3.31 (3.16-3.42)	2.92 (2.73-3.10)	2.87 (2.78-2.97)	2.92 (2.81-3.04)	3.19 (3.08-3.31)
	Cancer Detection Rate	4.08 (3.95-4.22)	4.08 (3.92-4.25)	4.22 (4.08-4.35)	4.08 (3.84-4.28)	4.66 (4.52-4.79)	4.08 (3.95-4.22)	4.34 (4.22-4.46)
	Abnormal interpretation rate	93.11 (92.58-93.61)	107.61 (106.90-108.23)	105.29 (104.78-105.87)	155.65 (154.36-156.57)	157.79 (157.27-158.51)	118.44 (117.90-119.16)	117.31 (116.69-117.94)
	True positives	4227	2838	4274	1708	4895	4432	4491
	True negatives	945942	629051	942686	360056	880442	966885	923143
	False positives	81468	61058	84724	55409	146968	111058	104267
	False negatives	3565	2026	3518	1220	2897	3166	3301
	Sensitivity	0.54 (0.53-0.55)	0.58 (0.57-0.60)	0.55 (0.56-0.58)	0.58 (0.57-0.60)	0.63 (0.58-0.64)	0.58 (0.57-0.60)	0.58 (0.57-0.59)
**AI-3**	Specificity	0.92 (0.91-0.93)	0.91 (0.90-0.92)	0.92 (0.92-0.94)	0.87 (0.85-0.88)	0.86 (0.81-0.88)	0.89 (0.88-0.90)	0.90 (0.89-0.91)
	Pos Pred Value	0.05 (0.05-0.05)	0.04 (0.04-0.05)	0.05 (0.05-0.05)	0.03 (0.03-0.03)	0.03 (0.03-0.03)	0.04 (0.04-0.04)	0.04 (0.04-0.04)
	False Positive Rate	78.70 (78.18-79.27)	87.86 (87.22-88.68)	81.84 (81.39-82.34)	132.43 (131.57-133.60)	141.97 (141.21-142.72)	102.31 (101.73-103.06)	100.72 (100.30-101.34)
	False Negative Rate	3.44 (3.34-3.53)	2.92 (2.82-3.07)	3.40 (3.28-3.51)	2.92 (2.77-3.09)	2.80 (2.72-2.90)	2.92 (2.82-3.07)	3.19 (3.09-3.32)
	Cancer Detection Rate	4.08 (3.97-4.22)	4.08 (3.88-4.25)	4.13 (3.97-4.26)	4.08 (3.91-4.26)	4.73 (4.61-4.86)	4.08 (3.96-4.20)	4.34 (4.19-4.44)
	Abnormal interpretation rate	82.76 (82.33-83.37)	91.94 (91.39-92.74)	86.00 (85.45-86.47)	136.48 (135.61-137.56)	146.68 (146.12-147.29)	106.41 (105.88-106.93)	105.11 (104.50-105.55)

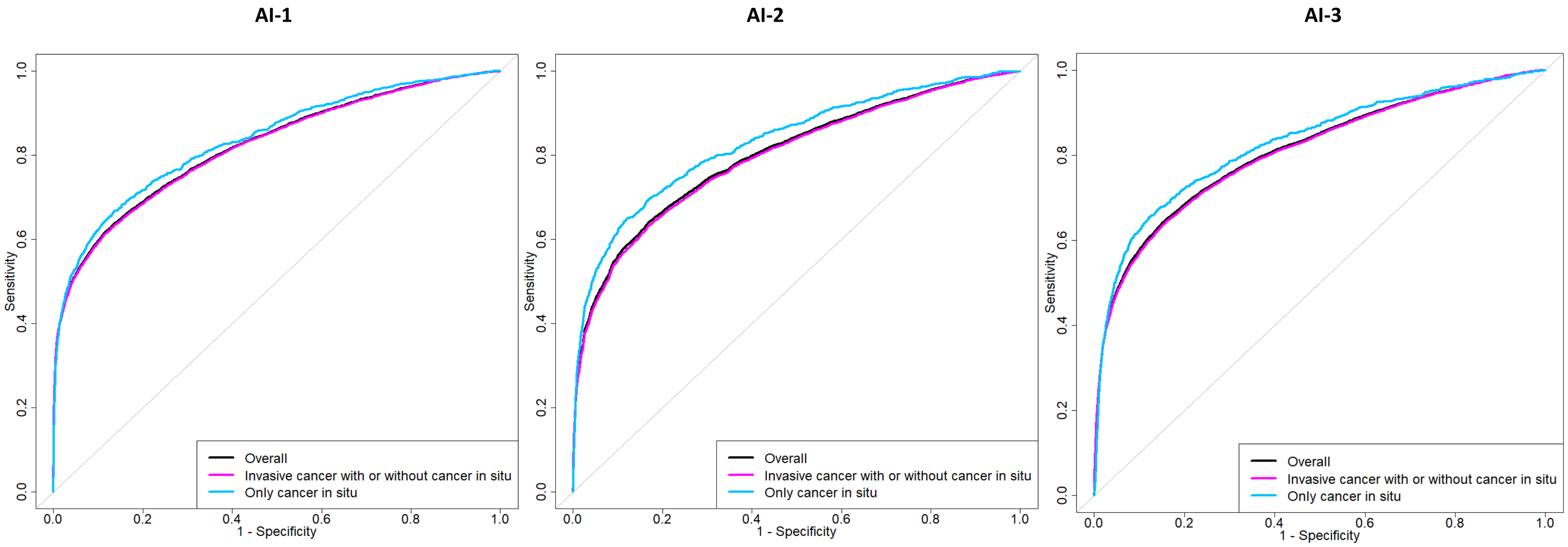

Supplementary Figure 11: ROC of Cancer characteristics. *ROC curve for AI systems performance on invasive (with or without cancer in situ) and in situ cancer diagnosis data compared to the* representative *data.*

AI-1	Overall 1027410 (7457)	Invasive cancer with or without cancer in situ 1027410 (6462)	Only cancer in situ 1027410 (995)
Overall 1027410 (7457)	/	0.00 (0.52)	-0.02 (-1.78)
Invasive cancer with or without cancer in situ 1027410 (6462)	0.00 (-0.52)	/	-0.02 (-2.03)
Only cancer in situ 1027410 (995)	0.02 (1.78)	0.03 (2.03)	/

P-value = *<0.05, **<0.01, *** <0.001
Rows and Columns: Categories with number of controls (cases).
Cells: Delta AUC (D-statistic)

AI-2	Overall 1027410 (7457)	Invasive cancer with or without cancer in situ 1027410 (6462)	Only cancer in situ 1027410 (995)
Overall 1027410 (7457)	/	0.01 (1.09)	-0.03 (-3.84) ***
Invasive cancer with or without cancer in situ 1027410 (6462)	-0.01 (-1.09)	/	-0.04 (-4.37) ***
Only cancer in situ 1027410 (995)	0.03 (3.84) ***	0.04 (4.37) ***	/

P-value = *<0.05, **<0.01, *** <0.001
Rows and Columns: Categories with number of controls (cases).
Cells: Delta AUC (D-statistic)

AI-3	Overall 1027410 (7457)	Invasive cancer with or without cancer in situ 1027410 (6462)	Only cancer in situ 1027410 (995)
Overall 1027410 (7457)	/	0.01 (0.66)	-0.02 (-2.24)
Invasive cancer with or without cancer in situ 1027410 (6462)	-0.01 (-0.66)	/	-0.03 (-2.56) *
Only cancer in situ 1027410 (995)	0.02 (2.24)	0.03 (2.56) *	/

P-value = *<0.05, **<0.01, *** <0.001
Rows and Columns: Categories with number of controls (cases).
Cells: Delta AUC (D-statistic)

Legend

Delta AUC	
(0.10 , ∞)	
(0.05 , 0.10]	
(0.01 , 0.05]	
[0.01 , -0-01]	
(-0.01 , -0.05]	
(-0.05 , -0.10]	
(-0.10 , ∞)	

( ) denotes excluded end points and [ ] to denote included end points

Supplementary Figure 12: Cancer characteristics – AUROC comparison. *DeLong analysis to compare the AUC values for the different Cancer diagnosis, invasive (with or without cancer in situ), in situ and the representative dataset.*

Supplementary Table 6: Cancer characteristics. *Accuracy metrics to demonstrate the impact of using cancer diagnosis specific data, either invasive (with or without cancer in situ) or in situ, to calibrate the AI threshold and then applying these thresholds to the representative dataset or cancer specific diagnosis dataset compared to calibrating the representative or cancer diagnosis specific dataset to double radiologists' cancer detection rat.*

AI System	Accuracy Metrics	Applying the Representative data threshold			Applying the Invasive cancer data threshold			Applying the Insitu cancer data threshold		
		Representative data (95%CI)	Invasive cancer data (95%CI)	Insitu cancer data (95%CI)	Invasive cancer data (95%CI)	Representative data (95%CI)	Insitu cancer data (95%CI)	Insitu cancer data (95%CI)	Representative data (95%CI)	Invasive cancer data (95%CI)
AI-1	True positives	4227	3497	563	3769	4560	611	580	4330	3580
	True negatives	959843	856239	131828	831002	931574	127949	130747	951990	849220
	False positives	67567	60275	9307	85512	95836	13186	10388	75420	67294
	False negatives	3565	2965	432	2693	3232	384	415	3462	2882
	Sensitivity	0.54 (0.53-0.55)	0.54 (0.53-0.56)	0.56 (0.53-0.57)	0.58 (0.57-0.60)	0.58 (0.57-0.59)	0.61 (0.57-0.60)	0.58 (0.56-0.61)	0.56 (0.55-0.61)	0.55 (0.54-0.62)
	Specificity	0.93 (0.92-0.94)	0.93 (0.92-0.94)	0.93 (0.91-0.93)	0.91 (0.90-0.91)	0.90 (0.89-0.91)	0.91 (0.87-0.89)	0.93 (0.91-0.94)	0.93 (0.93-0.95)	0.92 (0.92-0.95)
	Pos Pred Value	0.06 (0.06-0.06)	0.06 (0.05-0.06)	0.06 (0.05-0.06)	0.04 (0.04-0.04)	0.05 (0.04-0.05)	0.04 (0.04-0.05)	0.05 (0.05-0.06)	0.05 (0.05-0.06)	0.05 (0.05-0.05)
	False Positive Rate	65.27 (64.91-65.76)	65.31 (64.90-65.73)	65.48 (64.03-67.00)	92.65 (92.06-93.33)	92.58 (92.09-93.17)	92.77 (91.17-94.16)	73.09 (71.72-74.39)	72.86 (72.32-73.29)	72.91 (72.22-73.53)
	False Negative Rate	3.44 (3.34-3.56)	3.21 (3.10-3.31)	3.04 (2.76-3.36)	2.92 (2.79-3.06)	3.12 (2.99-3.25)	2.70 (2.46-2.99)	2.92 (2.57-3.21)	3.34 (3.25-3.44)	3.12 (2.98-3.22)
	Cancer Detection Rate	4.08 (3.97-4.20)	3.79 (3.67-3.91)	3.96 (3.62-4.29)	4.08 (3.96-4.19)	4.41 (4.29-4.56)	4.30 (3.96-4.57)	4.08 (3.78-4.42)	4.18 (4.04-4.29)	3.88 (3.75-4.01)
	Abnormal interpretation rate	69.34 (68.75-69.78)	69.08 (68.60-69.68)	69.55 (67.76-70.56)	96.75 (96.16-97.39)	97.04 (96.28-97.70)	97.08 (95.24-98.79)	77.24 (75.59-78.75)	77.05 (76.41-77.70)	76.78 (76.20-77.52)
AI-2	True positives	4227	3468	600	3761	4589	649	580	4039	3304
	True negatives	935261	834319	128490	800490	897416	123291	129603	943471	841645
	False positives	92149	82195	12645	116024	129994	17844	11532	83939	74869
	False negatives	3565	2994	395	2701	3203	346	415	3753	3158
	Sensitivity	0.54 (0.53-0.55)	0.54 (0.53-0.55)	0.60 (0.55-0.61)	0.58 (0.57-0.59)	0.59 (0.57-0.59)	0.65 (0.59-0.66)	0.58 (0.55-0.62)	0.52 (0.56-0.61)	0.51 (0.50-0.655
	Specificity	0.91 (0.90-0.91)	0.91 (0.90-0.92)	0.91 (0.87-0.92)	0.87 (0.86-0.89)	0.87 (0.86-0.88)	0.87 (0.80-0.89)	0.92 (0.91-0.94)	0.92 (0.91-0.96)	0.92 (0.90-0.94)
	Pos Pred Value	0.04 (0.04-0.05)	0.04 (0.04-0.04)	0.05 (0.04-0.05)	0.03 (0.03-0.03)	0.03 (0.03-0.04)	0.04 (0.03-0.04)	0.05 (0.04-0.05)	0.05 (0.05-0.05)	0.04 (0.04-0.04)
	False Positive Rate	89.02 (88.42-89.64)	89.05 (88.45-89.72)	88.97 (87.26-90.43)	125.71 (124.90-126.68)	125.57 (125.03-126.35)	125.55 (123.48-127.49)	81.14 (79.55-82.53)	81.09 (80.49-81.61)	81.12 (80.51-81.66)
	False Negative Rate	3.44 (3.35-3.57)	3.24 (3.15-3.38)	2.78 (2.57-3.03)	2.93 (2.80-3.08)	3.09 (2.98-3.23)	2.43 (2.17-2.72)	2.92 (2.62-3.18)	3.63 (3.51-3.73)	3.42 (3.30-3.54)
	Cancer Detection Rate	4.08 (3.96-4.21)	3.76 (3.63-3.88)	4.22 (3.86-4.50)	4.07 (3.92-4.19)	4.43 (4.29-4.54)	4.57 (4.23-4.98)	4.08 (3.75-4.48)	3.90 (3.79-4.01)	3.58 (3.47-3.70)
	Abnormal interpretation rate	93.13 (92.37-93.87)	92.78 (92.19-93.46)	93.15 (91.38-95.08)	129.73 (129.02-130.54)	129.99 (129.24-130.72)	130.13 (128.18-131.54)	85.13 (83.52-86.41)	85.01 (84.39-85.48)	84.70 (84.09-85.26)
AI-3	True positives	4227	3483	592	3769	4572	634	580	4163	3431
	True negatives	945942	843807	129921	816192	914993	125678	130430	949637	847118
	False positives	81468	72707	11214	100322	112417	15457	10705	77773	69396
	False negatives	3565	2979	403	2693	3220	361	415	3629	3031
	Sensitivity	0.54 (0.53-0.56)	0.54 (0.53-0.55)	0.59 (0.55-0.60)	0.58 (0.57-0.59)	0.59 (0.57-0.60)	0.64 (0.59-0.65)	0.58 (0.55-0.60)	0.53 (0.55-0.61)	0.53 (0.52-0.56)
	Specificity	0.92 (0.91-0.93)	0.92 (0.91-0.93)	0.92 (0.89-0.93)	0.89 (0.88-0.90)	0.89 (0.88-0.90)	0.89 (0.85-0.90)	0.92 (0.91-0.94)	0.92 (0.91-0.96)	0.92 (0.91-0.95)
	Pos Pred Value	0.05 (0.05-0.05)	0.04 (0.04-0.05)	0.05 (0.05-0.05)	0.04 (0.04-0.04)	0.04 (0.04-0.04)	0.04 (0.04-0.04)	0.05 (0.05-0.06)	0.05 (0.05-0.05)	0.05 (0.05-0.05)
	False Positive Rate	78.70 (78.24-79.25)	78.78 (78.11-79.34)	78.90 (77.39-80.60)	108.69 (107.95-109.34)	108.59 (108.05-109.32)	108.75 (106.87-110.87)	75.32 (73.74-76.73)	75.13 (74.64-75.52)	75.19 (74.58-75.74)
	False Negative Rate	3.44 (3.31-3.55)	3.23 (3.12-3.36)	2.84 (2.61-3.17)	2.92 (2.80-3.03)	3.11 (3.01-3.24)	2.54 (2.31-2.89)	2.92 (2.65-3.22)	3.51 (3.39-3.63)	3.28 (3.19-3.40)
	Cancer Detection Rate	4.08 (3.97-4.20)	3.77 (3.65-3.94)	4.17 (3.83-4.47)	4.08 (3.94-4.21)	4.42 (4.31-4.57)	4.46 (4.12-4.87)	4.08 (3.81-4.48)	4.02 (3.88-4.16)	3.72 (3.60-3.89)
	Abnormal interpretation rate	82.77 (82.19-83.39)	82.55 (82.02-83.11)	83.07 (81.37-84.55)	112.79 (112.16-113.37)	113.03 (112.47-113.68)	113.21 (111.52-114.99)	79.31 (78.33-80.74)	79.15 (78.57-79.65)	78.89 (78.21-79.71)

AI-1	All Manufacturers 1024265 (7774)	Philips 386651 (2725)	GE 716354 (5049)
All Manufacturers 1024265 (7774)	/	-0.04 (-7.82) ***	0.03 (5.62) ***
Philips 386651 (2725)	0.04 (7.82) ***	/	0.07 (11.72) ***
GE 716354 (5049)	-0.03 (-5.62) ***	-0.07 (-11.72) ***	/

P-value = *<0.05, **<0.01, *** <0.001
Rows and Columns: Categories with number of controls (cases).
Cells: Delta AUC (D-statistic)

AI-2	All Manufacturers 1024265 (7774)	Philips 386651 (2725)	GE 716354 (5049)
All Manufacturers 1024265 (7774)	/	-0.04 (-8.81) ***	0.02 (4.06) ***
Philips 386651 (2725)	0.04 (8.81) ***	/	0.06 (11.38) ***
GE 716354 (5049)	-0.02 (-4.06) ***	-0.06 (-11.38) ***	/

P-value = *<0.05, **<0.01, *** <0.001
Rows and Columns: Categories with number of controls (cases).
Cells: Delta AUC (D-statistic)

AI-3	All Manufacturers 1024265 (7774)	Philips 386651 (2725)	GE 716354 (5049)
All Manufacturers 1024265 (7774)	/	-0.06 (-12.16) ***	0.04 (7.08) ***
Philips 386651 (2725)	0.06 (12.16) ***	/	0.10 (16.96) ***
GE 716354 (5049)	-0.04 (-7.08) ***	-0.10 (-16.96) ***	/

P-value = *<0.05, **<0.01, *** <0.001
Rows and Columns: Categories with number of controls (cases).
Cells: Delta AUC (D-statistic)

Legend	
Delta AUC	
(0.10 , ∞)	
(0.05 , 0.10]	
(0.01 , 0.05]	
[0.01 , -0-01]	
(-0.01 , -0.05]	
(-0.05 , -0.10]	
(-0.10 , ∞)	

( ) denotes excluded end points and [ ] to denote included end points

Supplementary Figure 13: Image acquisition on Philips and GE – AUROC comparison. *DeLong analysis to compare the AUC values for the different imaging equipment manufacturers compared to the overall representative dataset including all manufacturers.*

AI-1	All Manufacturers 1024265 (7774)	Phillips cases GE controls 819869 (2725)	GE cases Philips controls 204396 (5049)
All Manufacturers 1024265 (7774)	/	-0.08 (-19.36) ***	0.10 (18.6) ***
Phillips cases GE controls 819869 (2725)	0.08 (19.36) ***	/	0.18 (32.9) ***
GE cases Philips controls 204396 (5049)	-0.10 (-18.6) ***	-0.18 (-32.9) ***	/

P-value = *<0.05, **<0.01, *** <0.001
Rows and Columns: Categories with number of controls (cases).
Cells: Delta AUC (D-statistic)

AI-2	All Manufacturers 1024265 (7774)	Phillips cases GE controls 819869 (2725)	GE cases Philips controls 204396 (5049)
All Manufacturers 1024265 (7774)	/	-0.01 (-3.37) **	-0.01 (-2.51) *
Phillips cases GE controls 819869 (2725)	0.01 (3.37) **	/	0.00 (1.27)
GE cases Philips controls 204396 (5049)	0.01 (2.51) *	0.00 (-1.27)	/

P-value = *<0.05, **<0.01, *** <0.001
Rows and Columns: Categories with number of controls (cases).
Cells: Delta AUC (D-statistic)

AI-3	All Manufacturers 1024265 (7774)	Phillips cases GE controls 819869 (2725)	GE cases Philips controls 204396 (5049)
All Manufacturers 1024265 (7774)	/	-0.07 (-16.95) ***	0.07 (12.87) ***
Phillips cases GE controls 819869 (2725)	0.07 (16.95) ***	/	0.14 (26.03) ***
GE cases Philips controls 204396 (5049)	-0.07 (-12.87) ***	-0.14 (-26.03) ***	/

P-value = *<0.05, **<0.01, *** <0.001
Rows and Columns: Categories with number of controls (cases).
Cells: Delta AUC (D-statistic)

Legend

Delta AUC	
(0.10 , ∞)	
(0.05 , 0.10]	
(0.01 , 0.05]	
[0.01 , -0-01]	
(-0.01 , -0.05]	
(-0.05 , -0.10]	
(-0.10 , ∞)	

( ) denotes excluded end points and [ ] to denote included end points

Supplementary Figure 14: Image acquisition mixing Philips and GE – AUROC comparison. *DeLong analysis to compare the AUC values for the non- representative imaging equipment manufacturers data, where case-control selections from different manufacturers were compared to the overall representative dataset including all manufacturers.*

Supplementary Table 7: Image acquisition. *Accuracy metrics to demonstrate the impact of using specific imaging equipment manufacturers data to calibrate the AI threshold and applying the thresholds on other equipment manufacturers or the overall representative dataset containing all manufacturers compared to calibrating these datasets directly to the radiologists double reading cancer detection rate.*

AI System	Accuracy Metrics	Applying the Representative data threshold			Applying the GE only data threshold			Applying the Philips only data threshold		
		Representative data (95%CI)	GE data (95%CI)	Philips data (95%CI)	GE data (95%CI)	Representative data (95%CI)	Philips data (95%CI)	Philips data (95%CI)	Representative data (95%CI)	GE data (95%CI)
	True positives	4214	2391	1823	2946	5067	2121	1590	3654	2064
	True negatives	957182	674107	351211	618167	865354	298575	370394	991219	694936
	False positives	67083	42247	35440	98187	158911	88076	16257	33046	21418
	False negatives	3560	2658	902	2103	2707	604	1135	4120	2985
	Sensitivity	0.54 (0.53-0.55)	0.47 (0.45-0.52)	0.67 (0.65-0.69)	0.58 (0.57-0.60)	0.65 (0.61-0.67)	0.78 (0.72-0.80)	0.58 (0.56-0.60)	0.47 (0.45-0.49)	0.40 (0.38-0.43)
AI-1	Specificity	0.94 (0.93-0.94)	0.94 (0.94-0.97)	0.908 (0.89-0.92)	0.86 (0.85-0.87)	0.85 (0.80-0.88)	0.77 (0.75-0.79)	0.95 (0.95-0.96)	0.97 (0.97-0.99)	0.97 (0.96-0.99)
	Pos Pred Value	0.06 (0.06-0.06)	0.05 (0.05-0.06)	0.05 (0.05-0.05)	0.03 (0.03-0.03)	0.03 (0.03-0.03)	0.02 (0.02-0.03)	0.09 (0.09-0.09)	0.10 (0.10-0.10)	0.09 (0.08-0.09)
	False Positive Rate	65.00 (64.48-65.47)	58.56 (58.05-59.12)	91.02 (90.12-92.17)	136.11 (135.45-136.82)	153.98 (153.49-154.69)	226.19 (224.84-227.36)	41.75 (41.09-42.33)	32.02 (31.75-32.37)	29.69 (29.32-30.13)
	False Negative Rate	3.45 (3.34-3.57)	3.68 (3.54-3.84)	2.32 (2.15-2.45)	2.92 (2.76-3.07)	2.62 (2.50-2.72)	1.55 (1.43-1.67)	2.92 (2.76-3.11)	3.99 (3.87-4.11)	4.14 (4.02-4.30)
	Cancer Detection Rate	4.08 (3.96-4.23)	3.31 (3.21-3.46)	4.68 (4.43-4.91)	4.08 (3.89-4.25)	4.91 (4.78-5.05)	5.45 (5.19-5.70)	4.08 (3.90-4.35)	3.54 (3.39-3.68)	2.86 (2.75-3.01)
	Abnormal interpretation rate	69.07 (68.46-69.55)	61.85 (61.34-62.35)	95.78 (94.70-96.56)	140.25 (139.45-141.27)	158.91 (158.33-159.63)	231.73 (230.27-233.12)	45.76 (45.17-46.45)	35.52 (35.18-35.90)	32.54 (32.06-32.91)
	True positives	4214	2568	1646	2946	4704	1758	1590	4151	2561
	True negatives	932438	649222	358220	606995	878518	347580	362659	935555	649898
	False positives	91827	67132	28431	109359	145747	39071	23992	88710	66456
	False negatives	3560	2481	1079	2103	3070	967	1135	3623	2488
	Sensitivity	0.54 (0.53-0.55)	0.51 (0.50-0.53)	0.60 (0.58-0.62)	0.58 (0.57-0.60)	0.60 (0.58-0.62)	0.65 (0.62-0.68)	0.58 (0.57-0.60)	0.53 (0.50-0.58)	0.51 (0.50-0.52)
AI-2	Specificity	0.91 (0.90-0.92)	0.91 (0.90-0.93)	0.92 (0.90-0.94)	0.85 (0.83-0.86)	0.86 (0.4-0.88)	0.89 (0.88-0.92)	0.94 (0.93-0.94)	0.91 (0.87-0.95)	0.91 (0.90-0.93)
	Pos Pred Value	0.04 (0.04-0.05)	0.04 (0.04-0.04)	0.06 (0.05-0.06)	0.03 (0.03-0.03)	0.03 (0.03-0.03)	0.04 (0.04-0.05)	0.06 (0.06-0.07)	0.05 (0.04-0.05)	0.04 (0.03-0.04)
	False Positive Rate	88.98 (88.45-89.45)	93.06 (92.23-93.75)	73.02 (72.19-73.89)	151.59 (150.78-152.60)	141.22 (140.39-141.88)	100.34 (99.49-101.48)	61.62 (60.78-62.44)	85.96 (85.49-86.46)	92.12 (91.37-92.91)
	False Negative Rate	3.45 (3.30-3.58)	3.44 (3.29-3.60)	2.77 (2.58-2.97)	2.92 (2.79-3.03)	2.97 (2.86-3.07)	2.48 (2.33-2.65)	2.92 (2.72-3.07)	3.51 (3.41-3.63)	3.45 (3.30-3.58)
	Cancer Detection Rate	4.08 (3.94-4.22)	3.56 (3.43-3.71)	4.23 (4.03-4.46)	4.08 (3.92-4.22)	4.56 (4.40-4.69)	4.52 (4.26-4.71)	4.08 (3.88-4.28)	4.02 (3.90-4.17)	3.55 (3.39-3.71)
	Abnormal interpretation rate	93.06 (92.52-93.55)	96.61 (95.92-97.39)	77.25 (76.47-78.08)	155.62 (154.99-156.48)	145.75 (145.02-146.28)	104.86 (103.72-105.63)	65.73 (64.98-66.72)	89.97 (89.54-90.48)	95.61 (95.04-96.36)
	True positives	4214	2388	1826	2946	5060	2114	1590	3578	1988
	True negatives	943065	661143	352477	605639	856432	308760	372113	978907	683409
	False positives	81200	55211	34174	110715	167833	77891	14538	45358	32945
	False negatives	3560	2661	899	2103	2714	611	1135	4196	3061
	Sensitivity	0.54 (0.53-0.55)	0.47 (0.45-0.54)	0.67 (0.64-0.69)	0.58 (0.57-0.60)	0.65 (0.58-0.60)	0.77 (0.75-0.79)	0.58 (0.57-0.60)	0.46 (0.44-0.48)	0.39 (0.37-0.41)
AI-3	Specificity	0.92 (0.91-0.93)	0.92 (0.90-0.94)	0.91 (0.90-0.93)	0.85 (0.83-0.86)	0.84 (0.78-0.90)	0.79 (0.77-0.81)	0.96 (0.95-0.97)	0.96 (0.94-0.99)	0.95 (0.94-0.99)
	Pos Pred Value	0.05 (0.05-0.05)	0.04 (0.04-0.04)	0.05 (0.05-0.05)	0.03 (0.03-0.03)	0.03 (0.03-0.03)	0.03 (0.03-0.03)	0.10 (0.09-0.10)	0.07 (0.07-0.07)	0.06 (0.06-0.06)
	False Positive Rate	78.68 (78.17-79.21)	76.53 (75.79-77.08)	87.77 (86.78-88.60)	153.47 (152.85-154.39)	162.62 (161.93-163.38)	200.04 (198.71-201.50)	37.34 (36.68-38.06)	43.95 (43.52-44.32)	45.67 (45.22-46.13)
	False Negative Rate	3.45 (3.32-3.54)	3.69 (3.55-3.83)	2.31 (2.14-2.49)	2.92 (2.77-3.06)	2.63 (2.53-2.75)	1.57 (1.43-1.70)	2.92 (2.74-3.10)	4.07 (3.94-4.16)	4.24 (4.06-4.40)
	Cancer Detection Rate	4.08 (3.99-4.22)	3.31 (3.18-3.41)	4.69 (4.48-4.89)	4.08 (3.96-4.24)	4.90 (4.74-5.03)	5.43 (5.22-5.64)	4.08 (3.89-4.29)	3.47 (3.36-3.58)	2.76 (2.64-2.88)
	Abnormal interpretation rate	82.75 (82.15-83.28)	79.81 (79.19-80.59)	92.50 (91.37-93.68)	157.56 (156.61-158.37)	167.53 (166.86-168.20)	205.41 (204.07-206.44)	41.44 (40.78-42.06)	47.41 (47.08-47.86)	48.42 (47.82-48.93)

Supplementary Table 8: Image acquisition. *Accuracy metrics to demonstrate the impact of using data containing non-representative case-control selections from different imaging equipment manufacturers to calibrate the AI threshold and then applying the threshold to the overall representative data compared to calibrating the representative dataset to the radiologists double reading cancer detection rate.*

AI System	Accuracy Metrics	Representative data calibration (95%CI)	Applying the GE cases and Philips controls data threshold		Applying the GE controls and Philips cases data threshold	
			GE cases Philips controls data (95%CI)	Representative data (95%CI)	GE controls Philips cases data (95%CI)	Representative data (95%CI)
AI-1	True positives	4214	2946	5067	1523	3503
	True negatives	957182	553119	865354	361249	997297
	False positives	67083	163165	158911	9091	26968
	False negatives	3560	2103	2707	1202	4271
	Sensitivity	0.54 (0.53-0.55)	0.58 (0.57-0.60)	0.65 (0.59-0.69)	0.56 (0.54-0.57)	0.45 (0.43-0.47)
	Specificity	0.94 (0.93-0.94)	0.77 (0.75-0.79)	0.85 (0.81-0.89)	0.98 (0.97-0.98)	0.97 (0.95-0.99)
	Pos Pred Value	0.06 (0.06-0.06)	0.02 (0.02-0.02)	0.03 (0.03-0.03)	0.14 (0.13-0.15)	0.12 (0.11-0.12)
	False Positive Rate	65.00 (64.53-65.45)	226.20 (225.13-227.15)	153.98 (153.13-154.91)	24.37 (23.79-24.86)	26.13 (25.84-26.41)
	False Negative Rate	3.45 (3.34-3.55)	2.92 (2.80-3.01)	2.62 (2.52-2.72)	3.22 (3.03-3.41)	4.14 (4.02-4.25)
	Cancer Detection Rate	4.08 (3.94-4.20)	4.08 (3.92-4.25)	4.91 (4.76-5.05)	4.08 (3.89-4.33)	3.39 (3.30-3.52)
	Abnormal interpretation rate	69.08 (68.48-69.57)	230.19 (229.16-231.24)	158.88 (158.15-159.77)	28.45 (27.94-28.95)	29.55 (29.24-29.88)
AI-2	True positives	4214	2946	4704	1523	4053
	True negatives	932438	643922	878143	337146	939898
	False positives	91827	72362	146122	33194	84367
	False negatives	3560	2103	3070	1202	3721
	Sensitivity	0.54 (0.53-0.55)	0.58 (0.57-0.60)	0.61 (0.59-0.62)	0.56 (0.53-0.59)	0.52 (0.52-0.56)
	Specificity	0.91 (0.90-0.91)	0.89 (0.88-0.91)	0.86 (0.84-0.89)	0.91 (0.91-0.92)	0.92 (0.92-0.95)
	Pos Pred Value	0.04 (0.04-0.05)	0.04 (0.04-0.04)	0.03 (0.03-0.03)	0.04 (0.04-0.05)	0.05 (0.04-0.04)
	False Positive Rate	88.98 (88.29-89.33)	100.32 (99.61-100.99)	141.59 (140.75-142.41)	88.98 (88.18-89.88)	81.75 (81.34-82.29)
	False Negative Rate	3.45 (3.32-3.60)	2.92 (2.77-3.05)	2.98 (2.89-3.08)	3.22 (3.05-3.39)	3.61 (3.50-3.69)
	Cancer Detection Rate	4.08 (3.96-4.24)	4.08 (3.93-4.25)	4.56 (4.45-4.69)	4.08 (3.86-4.26)	3.93 (3.80-4.06)
	Abnormal interpretation rate	93.13 (92.38-93.58)	104.40 (103.71-105.09)	146.16 (145.37-146.92)	93.01 (91.85-93.95)	85.68 (85.15-86.17)
AI-3	True positives	4214	2946	5060	1523	3439
	True negatives	943065	572101	856459	354997	983683
	False positives	81200	144183	167806	15343	40582
	False negatives	3560	2103	2714	1202	4335
	Sensitivity	0.54 (0.53-0.55)	0.58 (0.57-0.60)	0.65 (0.61-0.69)	0.56 (0.54-0.58)	0.44 (0.43-0.46)
	Specificity	0.92 (0.91-0.93)	0.80 (0.78-0.81)	0.84 (0.81-0.88)	0.96 (0.96-0.96)	0.96 (0.96-0.98)
	Pos Pred Value	0.05 (0.05-0.05)	0.02 (0.02-0.02)	0.03 (0.03-0.03)	0.09 (0.08-0.09)	0.08 (0.08-0.08)
	False Positive Rate	78.68 (78.29-79.33)	199.88 (198.68-201.22)	162.60 (161.69-163.24)	41.13 (40.40-41.89)	39.32 (39.01-39.73)
	False Negative Rate	3.45 (3.34-3.56)	2.92 (2.80-3.08)	2.63 (2.53-2.72)	3.22 (3.05-3.40)	4.20 (4.08-4.35)
	Cancer Detection Rate	4.08 (3.97-4.18)	4.08 (3.96-4.24)	4.90 (4.75-5.01)	4.08 (3.84-4.28)	3.33 (3.23-3.45)
	Abnormal interpretation rate	82.79 (82.17-83.33)	204.00 (203.05-204.87)	167.47 (166.67-168.18)	45.19 (44.40-45.92)	42.66 (42.21-43.03)

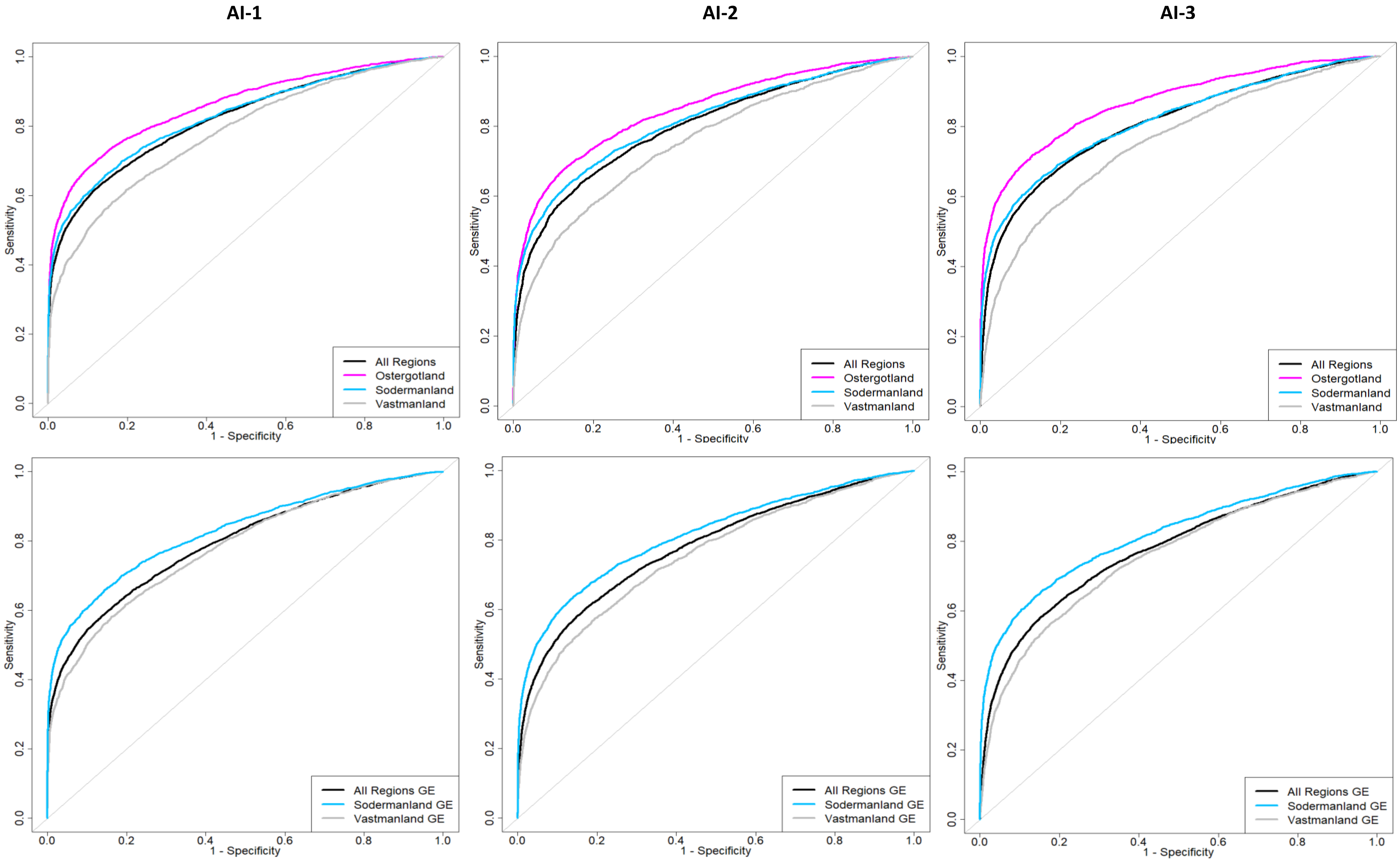

Supplementary Figure 15: ROC of Regional data. *ROC curve to compare the performance of AI systems on data from three different regions across Sweden (Östergötland, Södermanland and Västmanland), regardless of the imaging equipment manufacturer used* ***[top].*** *ROC for the performance of AI systems in the two regions (Södermanland and Västmanland) using GE imaging equipment manufacturer* ***[bottom].***

AI-1	All Regions 1027410 (7792)	Östergötland 206205 (2743)	Södermanland 257522 (2945)	Västmanland 562320 (2104)
All Regions 1027410 (7792)	/	-0.04 (-7.79) ***	-0.01 (-1.29)	0.04 (6.43) ***
Östergötland 206205 (2743)	0.04 (7.79) ***	/	0.03 (5.26) ***	0.08 (11.36) ***
Södermanland 257522 (2945)	0.01 (1.29)	-0.03 (-5.26) ***	/	0.05 (6.53) ***
Västmanland 562320 (2104)	-0.04 (-6.43) ***	-0.08 (-11.36) ***	-0.05 (-6.53) ***	/

P-value = *<0.05, **<0.01, *** <0.001
Rows and Columns: Categories with number of controls (cases).
Cells: Delta AUC (D-statistic)

AI-2	All Regions 1027410 (7792)	Östergötland 206205 (2743)	Sodermanland 257522 (2945)	Västmanland 562320 (2104)
All Regions 1027410 (7792)	/	-0.05 (-8.65) ***	-0.01 (-2.55)	0.05 (6.88) ***
Östergötland 206205 (2743)	0.05 (8.65) ***	/	0.04 (4.88) ***	0.10 (12.35) ***
Sodermanland 257522 (2945)	0.01 (2.55)	-0.04 (-4.88) ***	/	0.06 (7.86) ***
Västmanland 562320 (2104)	-0.05 (-6.88) ***	-0.10 (-12.35) ***	-0.06 (-7.86) ***	/

P-value = *<0.05, **<0.01, *** <0.001
Rows and Columns: Categories with number of controls (cases).
Cells: Delta AUC (D-statistic)

AI-3	All Regions 1027410 (7792)	Östergötland 206205 (2743)	Södermanland 257522 (2945)	Västmanland 562320 (2104)
All Regions 1027410 (7792)	/	-0.06 (-11.99) ***	-0.01 (-1.38)	0.05 (8.20) ***
Östergötland 206205 (2743)	0.06 (11.99) ***	/	0.05 (8.38) ***	0.11 (15.88) ***
Södermanland 257522 (2945)	0.01 (1.38)	-0.05 (-8.38) ***	/	0.06 (8.12) ***
Västmanland 562320 (2104)	-0.05 (-8.20) ***	-0.11 (-15.88) ***	-0.06 (-8.12) ***	/

P-value = *<0.05, **<0.01, *** <0.001
Rows and Columns: Categories with number of controls (cases).
Cells: Delta AUC (D-statistic)

Legend	
Delta AUC	
(0.10 , ∞)	
(0.05 , 0.10]	
(0.01 , 0.05]	
[0.01 , -0-01]	
(-0.01 , -0.05]	
(-0.05 , -0.10]	
(-0.10 , ∞)	

( ) denotes excluded end points and [ ] to denote included end points

Supplementary Figure 16: Regional data – AUROC comparison. *DeLong analysis to compare the AUC values for the datasets from the different regions (Östergötland, Södermanland and Västmanland), compared to the combined dataset covering all regions.*

Supplementary Table 9: *Overview of the screening participant characteristics (breast density, cancer diagnosis and age groups) across the three regions (Östergötland, Södermanland and Västmanland).*

Region	Category	Case-control	Total	A	B	C	D	Invasive	In situ	40-49	50-69	70+
Östergötland	Patients	Case	2328	219	1134	980	59	1945	271	393	1455	539
		Control	7451	1043	3426	2822	204	/	/	2320	4128	1034
	Exams	Case	2710	243	1291	1119	62	2259	318	451	1664	597
		Control	7632	1069	3481	2880	205	/	/	2375	4214	1057
Södermanland	Patients	Case	2045	154	1239	708	57	1740	235	353	1347	471
		Control	9045	956	5081	2859	278	/	/	2858	5122	1216
	Exams	Case	2933	184	1724	962	66	2501	344	468	1849	616
		Control	9654	981	5395	2989	289	/	/	2980	5425	1249
Västmanland	Patients	Case	1647	208	937	528	57	1304	262	286	1081	361
		Control	10828	2125	6392	3335	522	/	/	3171	7439	1897
	Exams	Case	2092	248	1155	628	62	1669	331	327	1345	420
		Control	21029	3403	11590	5294	755	/	/	3961	13782	3286

AI-1	All GE 819869 (5049)	Västmanland 562320 (2104)	Södermanland 257522 (2945)
All GE Regions 819869 (5049)	/	0.01 (2.19)	-0.04 (-5.59) ***
Västmanland 562320 (2104)	-0.01 (-2.19)	/	-0.05 (-6.53) ***
Södermanland 257522 (2945)	0.04 (-5.59) ***	0.05 (6.53) ***	/

P-value = *<0.05, **<0.01, *** <0.001
Rows and Columns: Categories with number of controls (cases).
Cells: Delta AUC (D-statistic)

AI-2	All GE 819869 (5049)	Västmanland 562320 (2104)	Sodermanland 257522 (2945)
All GE Regions 819869 (5049)	/	0.03 (3.77) ***	-0.03 (-5.48) ***
Västmanland 562320 (2104)	-0.03 (-3.77) ***	/	-0.06 (-7.86) ***
Södermanland 257522 (2945)	0.03 (5.48) ***	0.06 (7.86) ***	/

P-value = *<0.05, **<0.01, *** <0.001
Rows and Columns: Categories with number of controls (cases).
Cells: Delta AUC (D-statistic)

AI-3	All GE 819869 (5049)	Västmanland 562320 (2104)	Södermanland 257522 (2945)
All GE Regions 819869 (5049)	/	0.02 (2.89) *	-0.04 (-6.78) ***
Västmanland 562320 (2104)	-0.02 (-2.89) *	/	-0.06 (-8.12) ***
Södermanland 257522 (2945)	0.04 (6.78) ***	0.06 (8.12) ***	/

P-value = *<0.05, **<0.01, *** <0.001
Rows and Columns: Categories with number of controls (cases).
Cells: Delta AUC (D-statistic)

Legend

Delta AUC	
(0.10 , ∞)	
(0.05 , 0.10]	
(0.01 , 0.05]	
[0.01 , -0-01]	
(-0.01 , -0.05]	
(-0.05 , -0.10]	
(-0.10 , ∞)	

( ) denotes excluded end points and [ ] to denote included end points

Supplementary Figure 17: GE only Regional data – AUROC comparison. *DeLong analysis to compare the AUC values for the datasets from the two regions (Södermanland and Västmanland) using GE imaging equipment compared to the combined dataset covering both regions using GE imaging equipment.*

Supplementary Table 10: *Overview of the GE manufacturer models and GE software versions in the two regions (Södermanland, Västmanland) using GE equipment.*

GE	Model or software version	Södermanland		Västmanland	
		Case	Control	Case	Control
Model	Senograph DS ADS_32.10	0	0	5	23
	Senograph DS ADS_43.10.1	0	0	3	61
	Senograph DS VERSION ADS_53.10.1	0	0	15	221
	Senograph DS VERSION ADS_53.40	0	0	16	266
	Senographe Essential ADS_41.0	0	0	30	291
	Senographe Essential ADS_41.02	0	0	2	32
	Senographe Essential ADS_43.0	0	0	9	169
	Senographe Essential ADS_43.10.1	0	0	132	1181
	Senographe Essential VERSION ADS_53.10	338	1165	0	0
	Senographe Essential VERSION ADS_53.10.1.1	220	870	336	3307
	Senographe Essential VERSION ADS_53.30	41	128	1115	10954
	Senographe Essential VERSION ADS_53.40	247	822	0	0
	Senographe Essential VERSION ADS_54.11	1575	5394	0	0
	Senographe Essential VERSION ADS_55.20	0	0	78	2067
	Senographe Pristina	524	1275	363	2502
Software	1.5	322	720	152	625
	2.1.11	107	273	190	1269
	2.3.0	93	271	0	0
	3.2.44	0	0	21	608
	3.3.2	2	11	0	0
	Ads Application Package VERSION ADS_32.10	0	0	5	23
	Ads Application Package VERSION ADS_41.0	0	0	30	291
	Ads Application Package VERSION ADS_41.02	0	0	2	32
	Ads Application Package VERSION ADS_43.0	0	0	9	169
	Ads Application Package VERSION ADS_43.10.1	0	0	135	1242
	Ads Application Package VERSION ADS_53.10	338	1165	0	0
	Ads Application Package VERSION ADS_53.10.1.1	220	870	351	3528
	Ads Application Package VERSION ADS_53.30	41	128	0	0
	Ads Application Package VERSION ADS_53.40	247	822	1131	11225
	Ads Application Package VERSION ADS_54.11	1575	5394	0	0
	Ads Application Package VERSION ADS_55.20	0	0	78	2062

Supplementary Table 11: Upscaling methods and case-control ratio. *Accuracy metrics to comparison the radiologist's double reading performance on the cohort dataset (2017) compared to the upscaled and bootstrapped case-control dataset, limited to exam year 2017, for both 1:1 and 1:5 case-control ratios.*

Case-control ratio	Accuracy Metrics	Radiologists double reading cohort (95%CI)	Radiologists double reading not upscaled (95%CI)	Radiologists double reading upscaled (95%CI)	Radiologists double reading bootstrap (95%CI)
1:1	True positives	384	384	384	384
	True negatives	38238	820	37858	4137
	False positives	1793	46	2127	197
	False negatives	483	482	482	482
	Sensitivity	0.44 (0.41-0.48)	0.44 (0.41-0.48)	0.44 (0.41-0.48)	0.44 (0.28-0.61)
	Specificity	0.96 (0.95-0.96)	0.95 (0.93-0.96)	0.95 (0.95-0.95)	0.95 (0.94-0.96)
	Pos Pred Value	0.18 (0.16-0.19)	0.89 (0.86-0.92)	0.15 (0.14-0.17)	0.15 (0.09-0.22)
	False Positive Rate	43.84 (41.89-45.85)	26.56 (19.05-34.63)	52.07 (49.91-54.22)	52.04 (41.60-62.36)
	False Negative Rate	11.81 (10.78-12.86)	278.29 (256.93-299.64)	11.80 (10.77-12.90)	11.78 (6.93-17.32)
	Cancer Detection Rate	9.39 (8.46-10.34)	221.71 (202.66-241.92)	9.40 (8.47-10.33)	9.39 (5.20-13.86)
	Abnormal interpretation rate	53.22 (51.10-55.41)	248.31 (228.06-267.89)	61.49 (59.12-63.87)	61.46 (50.23-72.75)
1:5	True positives	384	384	384	384
	True negatives	38238	4137	38166	4137
	False positives	1793	197	1819	197
	False negatives	483	482	482	482
	Sensitivity	0.44 (0.41-0.48)	0.44 (0.41-0.48)	0.44 (0.41-0.48)	0.44 (0.35-0.54)
	Specificity	0.96 (0.95-0.96)	0.96 (0.95-0.96)	0.96 (0.95-0.96)	0.96 (0.95-0.96)
	Pos Pred Value	0.18 (0.16-0.19)	0.66 (0.62-0.70)	0.17 (0.16-0.19)	0.17 (0.13-0.22)
	False Positive Rate	43.84 (41.89-45.85)	37.89 (32.89-43.08)	44.53 (42.55-46.51)	44.50 (39.04-50.00)
	False Negative Rate	11.81 (10.78-12.86)	92.69 (84.81-100.77)	11.80 (10.77-12.83)	11.80 (9.04-14.81)
	Cancer Detection Rate	9.39 (8.46-10.34)	73.85 (66.73-80.96)	9.40 (8.47-10.36)	9.41 (6.73-12.12)
	Abnormal interpretation rate	53.22 (51.10-55.41)	111.74 (103.27-120.39)	53.94 (51.75-56.13)	53.88 (47.89-60.00)
